# Author Correction: Limits to the strain engineering of layered square-planar nickelate thin films

**DOI:** 10.1038/s41467-024-49984-6

**Published:** 2024-07-05

**Authors:** Dan Ferenc Segedin, Berit H. Goodge, Grace A. Pan, Qi Song, Harrison LaBollita, Myung-Chul Jung, Hesham El-Sherif, Spencer Doyle, Ari Turkiewicz, Nicole K. Taylor, Jarad A. Mason, Alpha T. N’Diaye, Hanjong Paik, Ismail El Baggari, Antia S. Botana, Lena F. Kourkoutis, Charles M. Brooks, Julia A. Mundy

**Affiliations:** 1 Department of Physics, Harvard University, Cambridge, MA USA; 2 School of Applied and Engineering Physics, Cornell University, Ithaca, NY USA; 3 Kavli Institute at Cornell for Nanoscale Science, Cornell University, Ithaca, NY USA; 4 Department of Physics, Arizona State University, Tempe, AZ USA; 5 The Rowland Institute, Harvard University, Cambridge, MA USA; 6 School of Engineering and Applied Science, Harvard University, Cambridge, MA USA; 7 Department of Chemistry and Chemical Biology, Harvard University, Cambridge, MA USA; 8 Advanced Light Source, Lawrence Berkeley National Laboratory, Berkeley, CA USA; 9 Platform for the Accelerated Realization, Analysis, and Discovery of Interface Materials (PARADIM), Cornell University, Ithaca, NY USA; 10 Present Address: School of Electrical and Computer Engineering, University of Oklahoma, Norman, OK USA

**Keywords:** Superconducting properties and materials, Superconducting properties and materials, Surfaces, interfaces and thin films

Correction to: *Nature Communications* 10.1038/s41467-023-37117-4, published online 16 March 2023

In the Acknowledgements section of this article the grant number relating to NSF was incorrectly given as DMR 2045826 and should have been DMR-2045826. The original article has been corrected.

