# Supplementary material for: Limits to the strain engineering of layered square-planar nickelate thin films
Source: Nat Commun. 2023 Mar 16;14:1468. doi: 10.1038/s41467-023-37117-4 (PMC10020545; doi:10.1038/s41467-023-37117-4)
Supplement: Supplementary file 1 — Supplementary Information [file 41467_2023_37117_MOESM1_ESM.pdf]

Supplementary Material for: Limits to the strain engineering of layered square-planar nickelate thin films

Dan Ferenc Segedin,[1,*] Berit H. Goodge,[2,3,*] Grace A. Pan,[1] Qi Song,[1] Harrison LaBollita,[4] Myung-Chul Jung,[4] Hesham El-Sherif,[5] Spencer Doyle,[1] Ari Turkiewicz,[1] Nicole K. Taylor,[6] Jarad A. Mason,[7] Alpha T. N'Diaye,[8] Hanjong Paik,[9,10] Ismail El Baggari,[5] Antia S. Botana,[4] Lena F. Kourkoutis,[2,3] Charles M. Brooks,[1] and Julia A. Mundy[1,6,†]

[1]*Department of Physics, Harvard University, Cambridge, MA, USA*
[2]*School of Applied and Engineering Physics, Cornell University, Ithaca, NY, USA*
[3]*Kavli Institute at Cornell for Nanoscale Science, Cornell University, Ithaca, NY, USA*
[4]*Department of Physics, Arizona State University, Tempe, AZ, USA*
[5]*The Rowland Institute, Harvard University, Cambridge, MA, USA*
[6]*School of Engineering and Applied Science, Harvard University, Cambridge, MA, USA*
[7]*Department of Chemistry and Chemical Biology, Harvard University, Cambridge, MA, USA*
[8]*Advanced Light Source, Lawrence Berkeley National Laboratory, Berkeley, CA, USA*
[9]*Platform for the Accelerated Realization, Analysis, and Discovery of Interface Materials (PARADIM), Cornell University, Ithaca, NY, USA*
[10]*School of Electrical and Computer Engineering, University of Oklahoma, Norman, OK, USA*

(Dated: February 28, 2023)

* These authors contributed equally to this work.

† mundy@fas.harvard.edu

Supplementary note 1: Electronic structure calculations **S4**
FIG. S1: Summary of strain-dependent electronic structure of $Nd_4Ni_3O_8$, $Nd_6Ni_5O_{12}$ . . . S4
FIG. S2: Band structures and DOS calculations of strained $Nd_4Ni_3O_8$ and $Nd_6Ni_5O_{12}$ . . . S6
FIG. S3: Schematic cartoon of strained $Nd_4Ni_3O_8$ fermiology . . . S7

Supplementary note 2: Strain-dependent defect formation of vertical rock salt faults **S8**
FIG. S4: Ruddlesden-Popper faults as strain-relieving mechanism . . . S9

Supplementary note 3: Strain-dependence of the $Nd_4Ni_3O_{10}$ Structure **S10**
FIG. S5: XRD of $Nd_4Ni_3O_{10}$ films on $NdGaO_3$ and $LaAlO_3$ . . . S10

Supplementary note 4: Reductions on $LaAlO_3$ **S11**
FIG. S6: Reductions of three consecutively synthesized $Nd_4Ni_3O_{10}$ / $LaAlO_3$ films. . . . S11
FIG. S7: Reduction of the $Nd_4Ni_3O_{10}$ / $LaAlO_3$ sample in the main text. . . . S12
FIG. S8: Over-reduction of $Nd_4Ni_3O_{10}$ / $LaAlO_3$ film . . . S12
FIG. S9: Reductions of three consecutively-synthesized $Nd_6Ni_5O_{16}$ / $LaAlO_3$ films . . . S13
FIG. S10: Reduction of $NdNiO_3$ / $LaAlO_3$ . . . S14
FIG. S11: Incremental reductions of $Nd_4Ni_3O_{10}$ / $LaAlO_3$ . . . S14
FIG. S12: Reciprocal space maps of films on $LaAlO_3$ . . . S15
FIG. S13: Oxygen K-edge spectra of $n = 3$ and $n = 5$ films before and after reduction . . . S16

Supplementary note 5: Reductions on $NdGaO_3$ **S17**
FIG. S14: Thickness-dependent reductions of $Nd_4Ni_3O_{10}$ / $NdGaO_3$ . . . S17
FIG. S15: X-ray and transport characterization of an incrementally reduced $Nd_4Ni_3O_{10}$ / $NdGaO_3$ film . . . S18
FIG. S16: Reductions of the ‘optimal’ $Nd_4Ni_3O_{10}$ / $NdGaO_3$ sample . . . S19
FIG. S17: Reductions of the 3% neodymium-poor $Nd_4Ni_3O_{10}$ / $NdGaO_3$ sample . . . S20
FIG. S18: Reductions of the 3% neodymium-rich $Nd_4Ni_3O_{10}$ / $NdGaO_3$ sample . . . S21
FIG. S19: Reductions of the 6% neodymium-poor $Nd_4Ni_3O_{10}$ / $NdGaO_3$ sample . . . S21
FIG. S20: Reductions of the 6% neodymium-rich $Nd_4Ni_3O_{10}$ / $NdGaO_3$ sample . . . S22

FIG. S21: XRD and resistance versus temperature measurements of three $Nd_4Ni_3O_{10}$ / $NdGaO_3$ films S23
FIG. S22: Reciprocal space maps of strained films on $NdGaO_3$ S24

Supplementary note 6: Atomic structure of the film-substrate interfaces S25
FIG. S23: Elemental mapping across the film-substrate interface on $SrTiO_3$ S25
FIG. S24: Elemental mapping across the film-substrate interface on $NdGaO_3$ S26
FIG. S25: Elemental mapping across the film-substrate interface on $LaAlO_3$ S27

Supplementary note 7: Structural characterization by scanning transmission electron microscopy S28
FIG. S26: STEM images and strain maps from Figure 5 in the main text S28
FIG. S27: STEM image and lattice orientation map from Figure 7 . . S29
FIG. S28: Full field-of-view HAADF- and ABF-STEM images from Figure 7 . . . . S30
FIG. S29: HAADF-STEM image of a canting defect in reduced $Nd_4Ni_3O_8$ film on $LaAlO_3$ S31
FIG. S30: Full field-of-view HAADF- and ABF-STEM images from Figure 9 . . . . S32

Supplementary note 8: Cation stoichiometry S33
FIG. S31: Structural characterization of $Nd_4Ni_3O_{10}$ and $Nd_4Ni_3O_8$ films on $NdGaO_3$ with varying neodymium content S34
FIG. S32: Electronic characterization of $Nd_4Ni_3O_{10}$ and $Nd_4Ni_3O_8$ films on $NdGaO_3$ with varying neodymium content S35
FIG. S33: Nickel L_3 edge XAS of $Nd_4Ni_3O_{10}$ and $Nd_4Ni_3O_8$ phases for films with varying neodymium content S36

References S37

SUPPLEMENTARY NOTE 1: ELECTRONIC STRUCTURE CALCULATIONS

We employ density functional theory (DFT)-based calculations to understand basic trends in the electronic structure of the trilayer ($Nd_4Ni_3O_8$) and five-layer ($Nd_6Ni_5O_{12}$) nickelates with in-plane strain. Our calculations were performed using the projector augmented (PAW) method as implemented in VASP [1] with the Perdew-Burke-Ernzerhof (PBE) version of the exchange correlation functional [2]. Note that we put the Nd($4f$) states in the pseudopotential core. For both materials, we used a $12 \times 12 \times 12$ $\mathbf{k}$-grid for the Brillouin zone integration. To model the effects of strain, we change the in-plane lattice constant, then optimize the atomic positions and out-of-plane lattice constants keeping the in-plane lattice constants fixed.

In the context of the cuprate superconductors, the charge-transfer energy ($\Delta_{\rm CT}$) and bandwidth of the $d_{x^2-y^2}$ bands are relevant parameters in connection to T_c. Estimates for both of these quantities can be derived from our electronic structure calculations. Figure S1(a) highlights how the charge-transfer energy (a measure of the hybridization between the Ni($3d$) and O($2p$) states) is influenced with strain. We estimate the charge-transfer energy, defined as $\Delta_{\rm CT} = E_d - E_p$ from the band centroids: $E_\alpha = \int dE\, g_\alpha(E)E / \int dE\, g_\alpha(E)$, where g_α is the partial density of states for orbital α and $\alpha \in$ [Ni-$d_{x^2-y^2}$, O-p_σ]. We find that compressive ($\varepsilon < 0$) strain increases the charge-transfer energy (pushing the Ni($3d$) and O($2p$) states apart), while tensile strain ($\varepsilon > 0$) strain decreases the charge-transfer energy (this is trend is also captured in Fig. S2(c,d)).

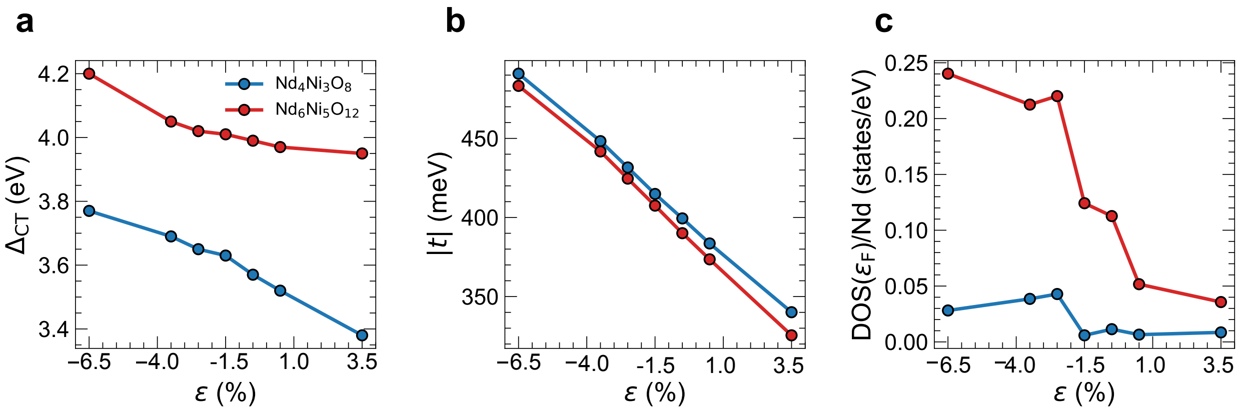

FIG. S1. Basic features of the electronic structure upon strain for $Nd_4Ni_3O_8$ (blue) and $Nd_6Ni_5O_{12}$ (red): (a) charge-transfer energy ($\Delta_{\rm CT}$), (b) nearest-neighbor hopping (t), and (c) the neodymium density of states at the Fermi level ($\varepsilon_{\rm F}$). The strain convention is the same one used in the main text: $\epsilon = (a_{sub} - a_{bulk})/a_{bulk}$. Note that -1.5% compressive strain corresponds to $Nd_4Ni_3O_{10}$ / $NdGaO_3$.

The nearest-neighbor hopping t between the Ni-$d_{x^2-y^2}$ orbitals is an important parameter as it sets the energy scale for the low-energy physics of the material. We estimate t by fitting

the DFT bands corresponding to the Ni-$d_{x^2-y^2}$ states to a tight-binding model on a square lattice,

$$\varepsilon(\mathbf{k}) = -\mu - 2t(\cos(k_x a) + \cos(k_y a)) - 4t'(\cos(k_x a)\cos(k_y a)) - 4t''(\cos(2k_x a) + \cos(2k_y a)), \tag{S1}$$

where μ is the chemical potential, t is the nearest-neighbor, t' is the next-nearest-neighbor, and t'' is the third-nearest-neighbor hoppping parameters. Figure S1(b) reveals the expected trend in the nearest-neighbor hopping. For in-plane compressive strain, the orbital overlap of Ni atoms is increased, which manifests in an overall increase in the $d_{x^2-y^2}$ bandwidth. Similarly, in-plane tensile strain decreases the bandwidth. Utilizing strain to control the overall bandwidth (and energy scale) has been suggested as a viable mechanism for enhancing T_c in the infinite-layer nickelates [3].

Finally, Fig. S1(c) highlights the evolution of the density of states (DOS) corresponding to the neodymium states at the Fermi level (ε_{F}). We observe that the neodymium DOS at ε_{F} is finite for $\epsilon < -1.5\%$, and approximately zero for $\epsilon > -1.5\%$. Thus strain has an influence on the magnitude of the neodymium DOS at ε_{F} similar to chemical doping [4].

Figure S2 displays a complete summary of the evolution of the band structure and density of states for the DFT calculations as function of strain from which the basic features that we describe above were derived. The overall trend in the charge-transfer energy can be visually seen from the evolution of the density of states in Fig. S2(c,d) and the decreasing bandwidth of the Ni-$d_{x^2-y^2}$ bands can be seen in Fig. S2(a,b). The trends in charge transfer energy and neodymium DOS at ε_{F} are schematically summarized in Fig. S3.

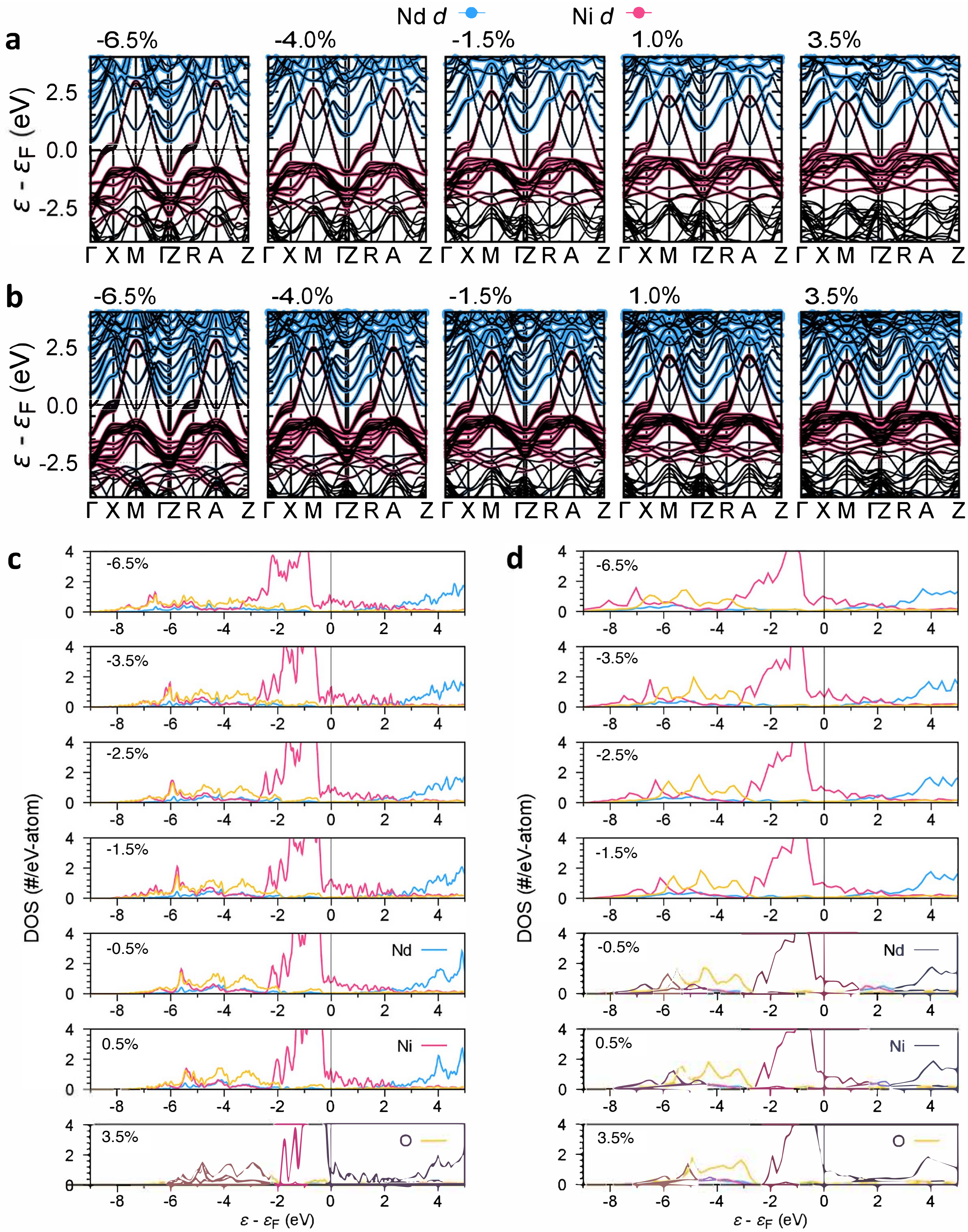

FIG. S2. DFT electronic structure for $Nd_4Ni_3O_8$ and $Nd_6Ni_5O_{12}$ upon applied strain. (Top panels) Paramagnetic band structures as function of strain for $Nd_4Ni_3O_8$ (a) and $Nd_6Ni_5O_{12}$ (b). (Bottom panels) Atom-resolved density of states (DOS) as a function of strain for $Nd_4Ni_3O_8$ (c) and $Nd_6Ni_5O_{12}$ (d).

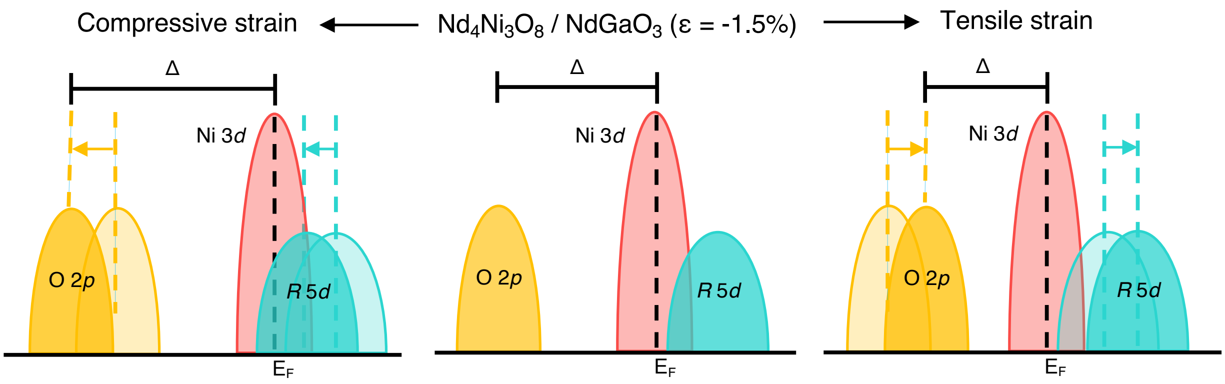

FIG. S3. Schematic cartoon highlighting the effect of compressive and tensile strain on the fermiology of $Nd_4Ni_3O_8$. We illustrate the influence of strain on the charge transfer energy Δ (Fig. S1(a)) and neodymium 5d density of states at ε_F (Fig. S1(c)). The effect of strain on the nickel 3d bandwidth is not shown (Fig. S1(b)) as it is a minor effect in comparison to the changes in charge transfer energy and neodymium DOS.

SUPPLEMENTARY NOTE 2: STRAIN-DEPENDENT DEFECT FORMATION OF VERTICAL ROCK SALT FAULTS

We estimate the relationship between the density of vertical rock salt faults density and effective lattice mismatch in nickelate thin films. We define the effective lattice mismatch as

$$\epsilon_{eff} = \frac{l_{sub} - l_{film}}{l_{film}}$$

where

$$l_{sub} = n_{sub} \cdot a_{sub}$$

$$l_{flim} = n_{film} \cdot a_{film} + n_{RP} \cdot d_{RP}$$

As illustrated in Fig. S4(a), n_{sub} and n_{film} denote the number of substrate and film unit cells, respectively, along the in-plane direction across a given lateral span. The substrate and film lattice constants are given by a_{sub} and a_{film}, respectively. The monolayer spacing between neodymium planes within a rock salt (RP) fault is denoted by d_{RP}, while n_{RP} represents the number of vertical RP faults in the same lateral span defined by n_{sub} and n_{film}. For a minimal fault density, the insertion of one vertical RP fault necessitates the formation of a second in order to maintain a coherent interface, as shown in Fig. S4(a). We thus set $n_{RP} = 2$. With the insertion of 2 vertical RP faults, two film monolayers (i.e. one unit cell) must be removed, giving $n_{film} = n_{sub} - 1$.

In Fig. S4(b), we plot the effective lattice mismatch

$$\epsilon_{eff}(n_{sub}) = \frac{(n_{sub} \cdot a_{sub}) - ((n_{sub} - 1) \cdot a_{film} + 2d_{RP})}{(n_{sub} - 1) \cdot a_{film} + 2d_{RP}}$$

for $Nd_4Ni_3O_{10}$ ($a_{film} = 3.826$Å) and $NdNiO_3$ ($a_{film} = 3.807$Å), with $d_{RP} = 2.76$Å[5] for the three substrates studied here: $SrTiO_3$ ($a_{sub} = 3.905$Å), $NdGaO_3$ ($a_{sub} = 3.858$Å), and $LaAlO_3$ ($a_{sub} = 3.790$Å). This calculation demonstrates how the insertion of vertical rock salt faults in nickelate films grown under tensile strain can effectively decrease the lattice mismatch. In fact, the insertion of approximately 4 and 9 vertical rock salt faults per 100 unit cells can effectively eliminate the lattice mismatch in $Nd_4Ni_3O_{10}$ grown on $NdGaO_3$ and $SrTiO_3$, respectively. For films grown under compressive strain, on the other hand, the insertion of vertical RP faults only further increases the magnitude of the lattice mismatch. Therefore, $Nd_4Ni_3O_{10}$ films grown on $NdGaO_3$ are more susceptible to the formation of tensile strain relieving RP faults than those grown on $LaAlO_3$, despite the fact that the magnitude of the lattice mismatch for both is approximately equal. Our calculation qualitatively describes the strain-dependent density of vertical RP faults we observe in Fig. 5 of the main text.

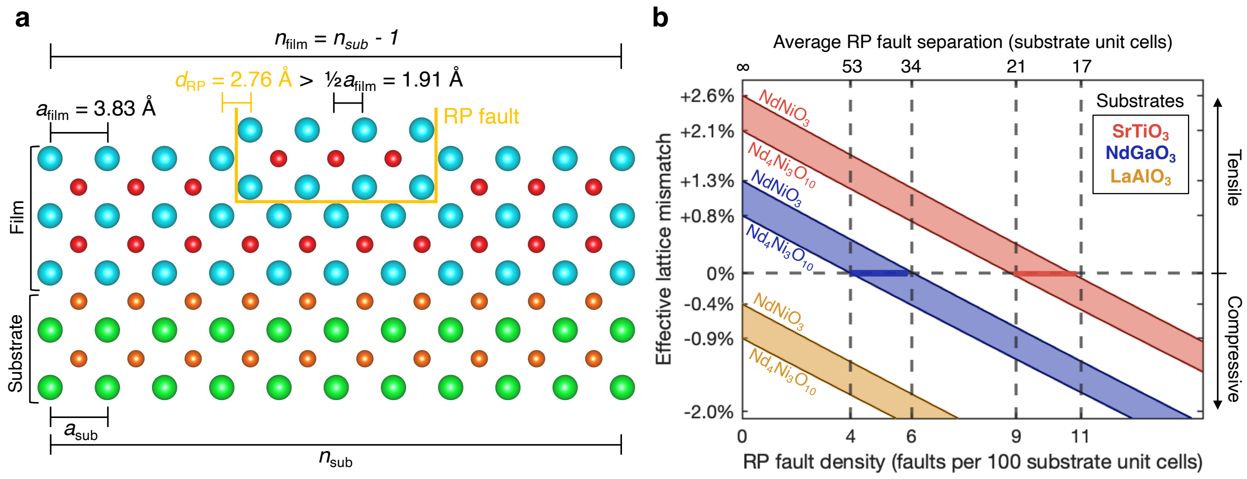

FIG. S4. (a) Schematic crystal structure of an epitaxial thin film with a rock salt fault, highlighted by the yellow line. The specified values for a_{film} and d_{RP} correspond to bulk $Nd_4Ni_3O_{10}$ [5]. (b) The effective lattice mismatch calculated for $Nd_4Ni_3O_{10}$ and $NdNiO_3$ thin films on $LaAlO_3$, $NdGaO_3$, and $SrTiO_3$ with varying density of vertical RP faults, plotted as a function of (top x-axis) the average fault separation (n_{sub}) and (bottom x-axis) the RP fault density per 100 substrate unit cells ($100 \cdot (2/n_{sub})$).

SUPPLEMENTARY NOTE 3: STRAIN-DEPENDENCE OF THE $Nd_4Ni_3O_{10}$ STRUCTURE

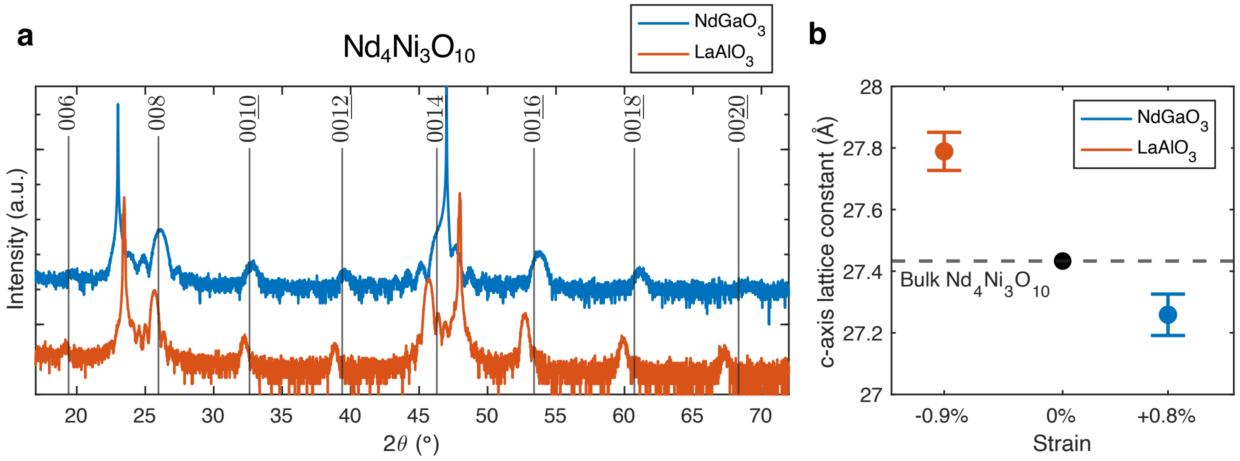

FIG. S5. (a) XRD scans of $Nd_4Ni_3O_{10}$ films grown on $NdGaO_3$ ($\epsilon = +0.8\%$) and $LaAlO_3$ ($\epsilon = -0.9\%$). (b) c-axis lattice constants calculated for the films shown in (a). The expansion (compression) of the c-axis lattice constant with compressive (tensile) strain demonstrates that the films are epitaxially strained to the susbtrate. The lattice constants are calculated via Nelson-Riley fits of the XRD peaks.

SUPPLEMENTARY NOTE 4: REDUCTIONS ON $LaAlO_3$

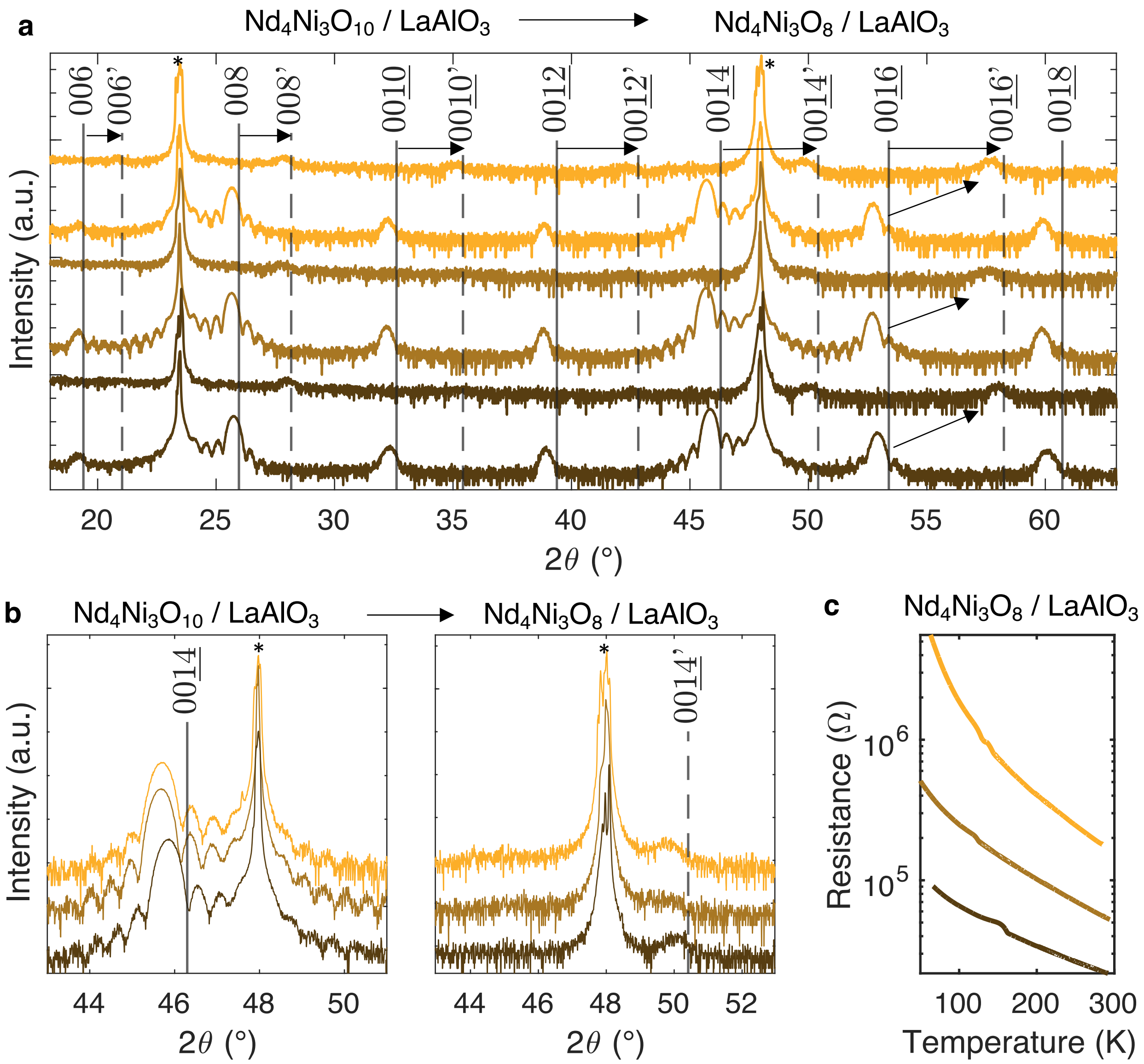

FIG. S6. Reductions of three consecutively synthesized $Nd_4Ni_3O_{10}$ / $LaAlO_3$ films. (a) XRD scans of the as-synthesized and reduced films. All films were reduced for 3 hours at 290°C. (b) Smaller field-of-view of the same XRD scans in (a). (c) Resistance vs. temperature measurements of the reduced samples in (a) and (b). The vertical solid and dashed lines denote the $00l$ peak positions of bulk $Nd_4Ni_3O_{10}$ and $Nd_4Ni_3O_8$, respectively. The primed indices distinguish the reduced square-planar phase from the as-synthesized Ruddlesden-Popper. The asterisks denote substrate reflections. Resistance measurements were taken using a home-built 'dipstick probe'.

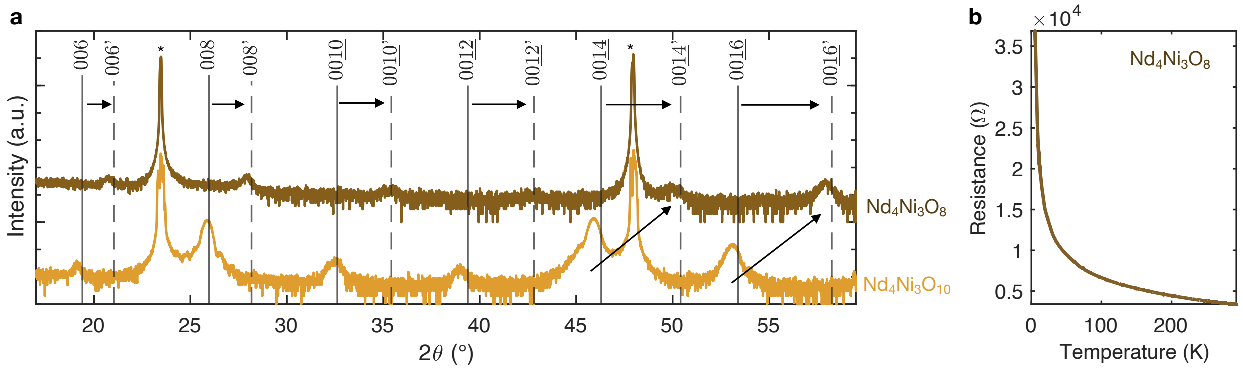

FIG. S7. Reduction of the $Nd_4Ni_3O_{10}$ / $LaAlO_3$ sample in Fig. 7 of the main text. (a) XRD scans of the as-synthesized $Nd_4Ni_3O_{10}$ and reduced $Nd_4Ni_3O_8$ film. (b) Resistance versus temperature of the reduced film in (a) taken using a home-built 'dipstick probe'.

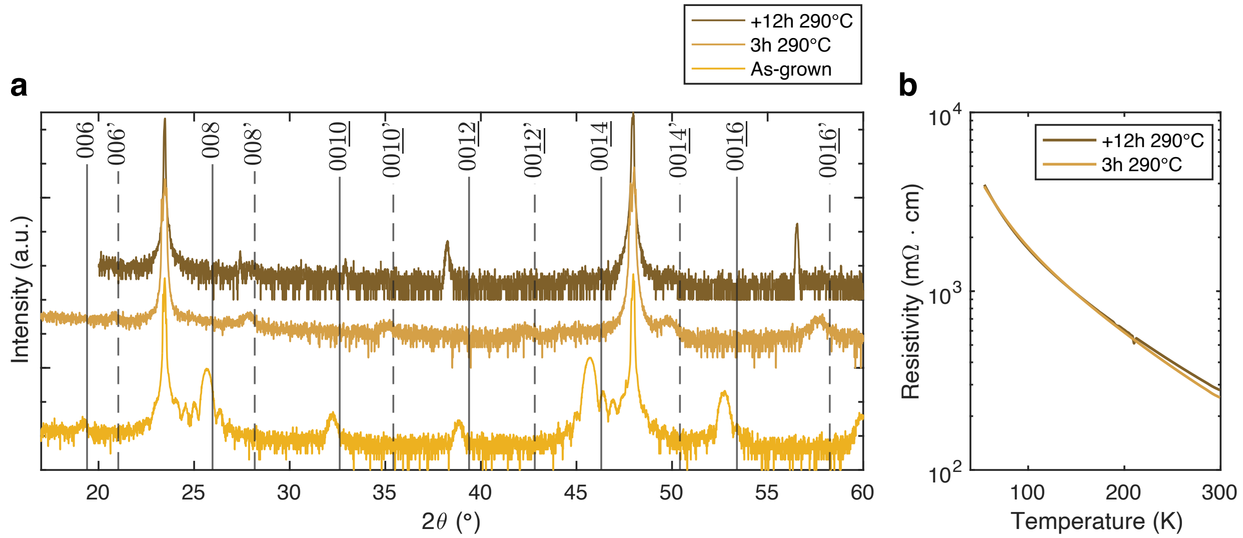

FIG. S8. (a) XRD scans of (from bottom to top) a $Nd_4Ni_3O_{10}$ / $LaAlO_3$ film as-grown, reduced for 3 hours at 290°C, and subsequently reduced for an additional 12 hours at 290°C. (b) Resistivity versus temperature measurements of the reduced films in (a). The reduced film peak intensities after 12 hours of reduction in (a) suggest structural degradation. The resistivity does not change after an additional 12 hours of reduction, although the x-ray peak intensities decrease.

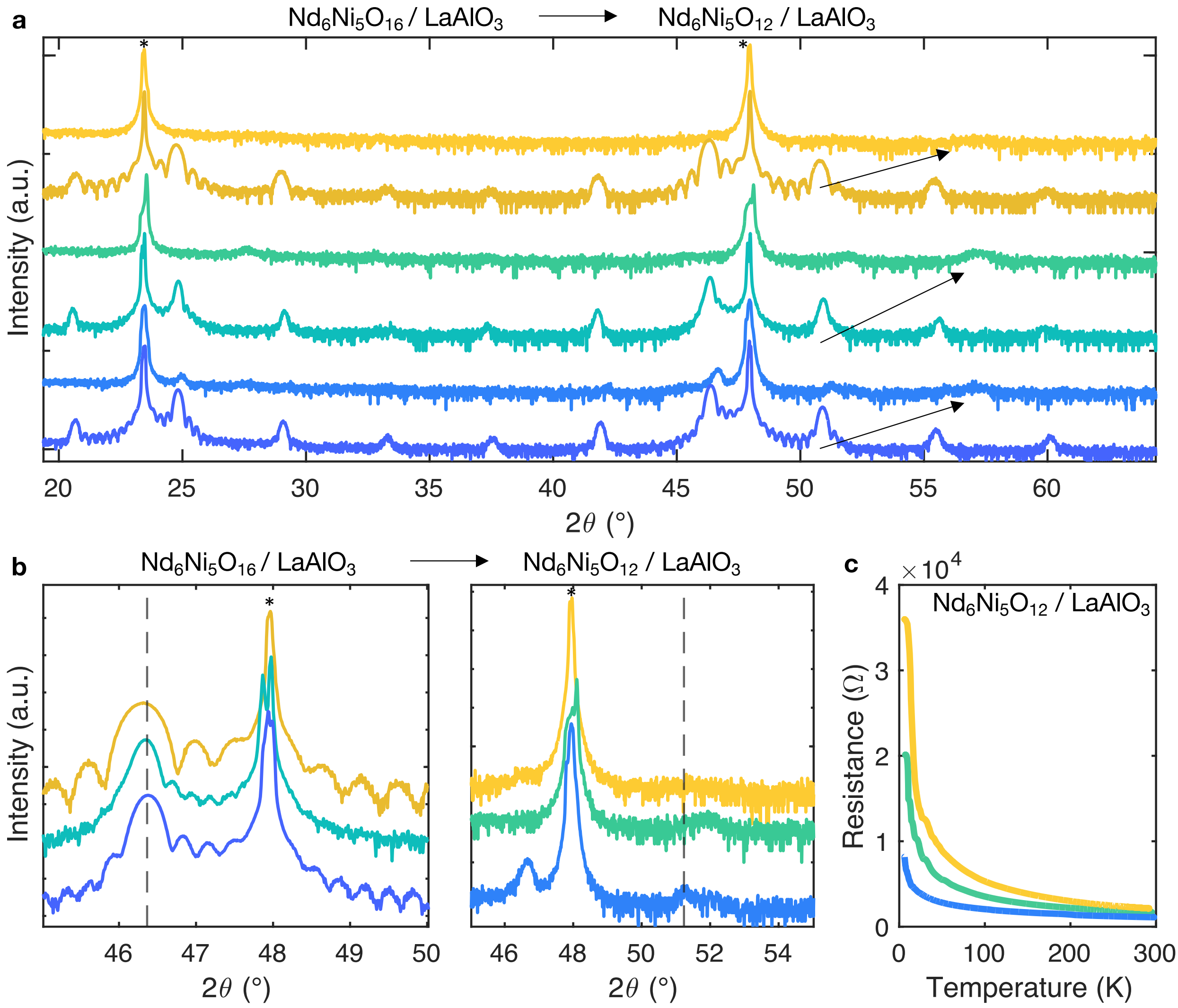

FIG. S9. Reductions of three consecutively-synthesized $Nd_6Ni_5O_{16}$ / $LaAlO_3$ films. (a) XRD scans of the as-synthesized and reduced films. (b) Zoom-in of the XRD scans in (a). The dotted lines are guides for comparing peak positions between the films. (c) Resistance vs. temperature measurements of the reduced films in (a). All films were reduced for 3 hours at 290°C. The asterisks denote substrate reflections. Resistance measurements were taken using a home-built 'dipstick probe'.

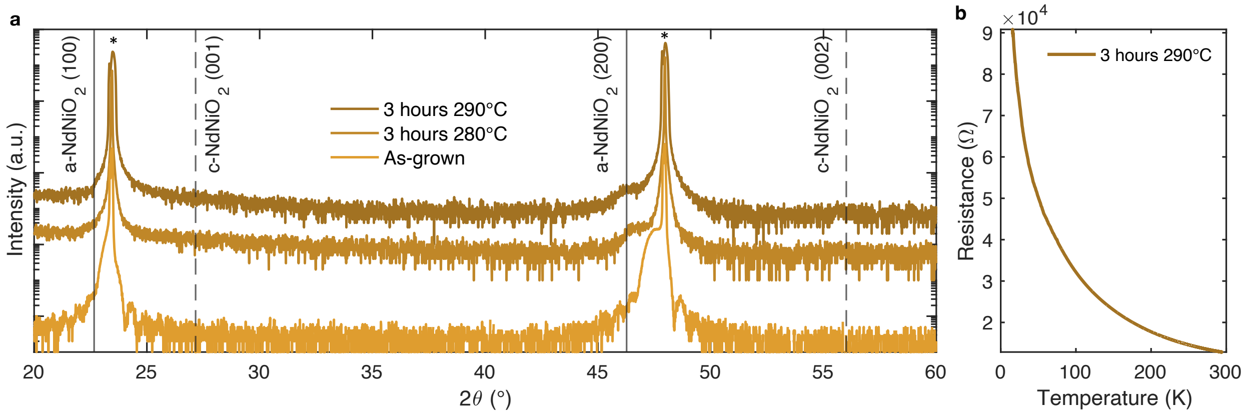

FIG. S10. Reduction of $NdNiO_3$ / $LaAlO_3$. (a) XRD scans of (i) the as-grown sample, then the same sample (but different wafers) reduced for (ii) 3 hours at 280°C and (iii) 3 hours at 290°C. (b) Resistance vs. temperature measurement of the film reduced for 3 hours at 290°C. The asterisks denote substrate reflections. Resistance measurements were taken using a home-built 'dipstick probe'.

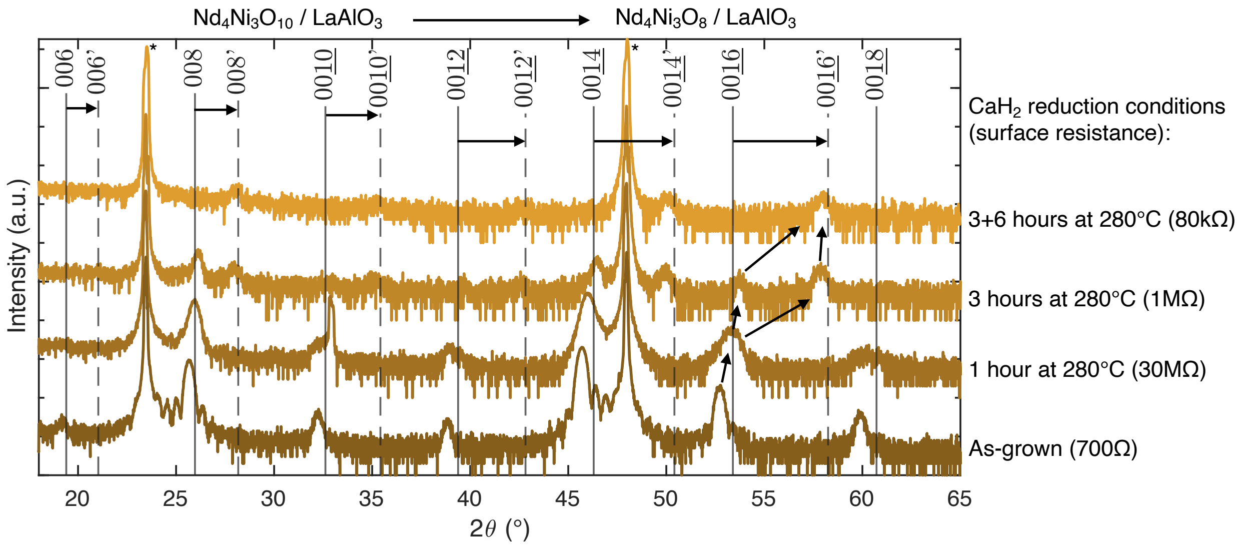

FIG. S11. Incremental reductions of $Nd_4Ni_3O_{10}$ / $LaAlO_3$. From bottom to top: (i) As-synthesized, then consecutive reductions at 280°C for (ii) 1 hours, (iii) 3 hours, and (iv) an additional 3 hours. The vertical solid and dotted lines denote $00l$ peak positions of bulk $Nd_4Ni_3O_{10}$ and $Nd_4Ni_3O_8$, respectively. The primed indices distinguish the reduced phase from the as-synthesized phase. Surface multi-meter resistances are indicated to the right of each XRD scan. The asterisks denote substrate reflections.

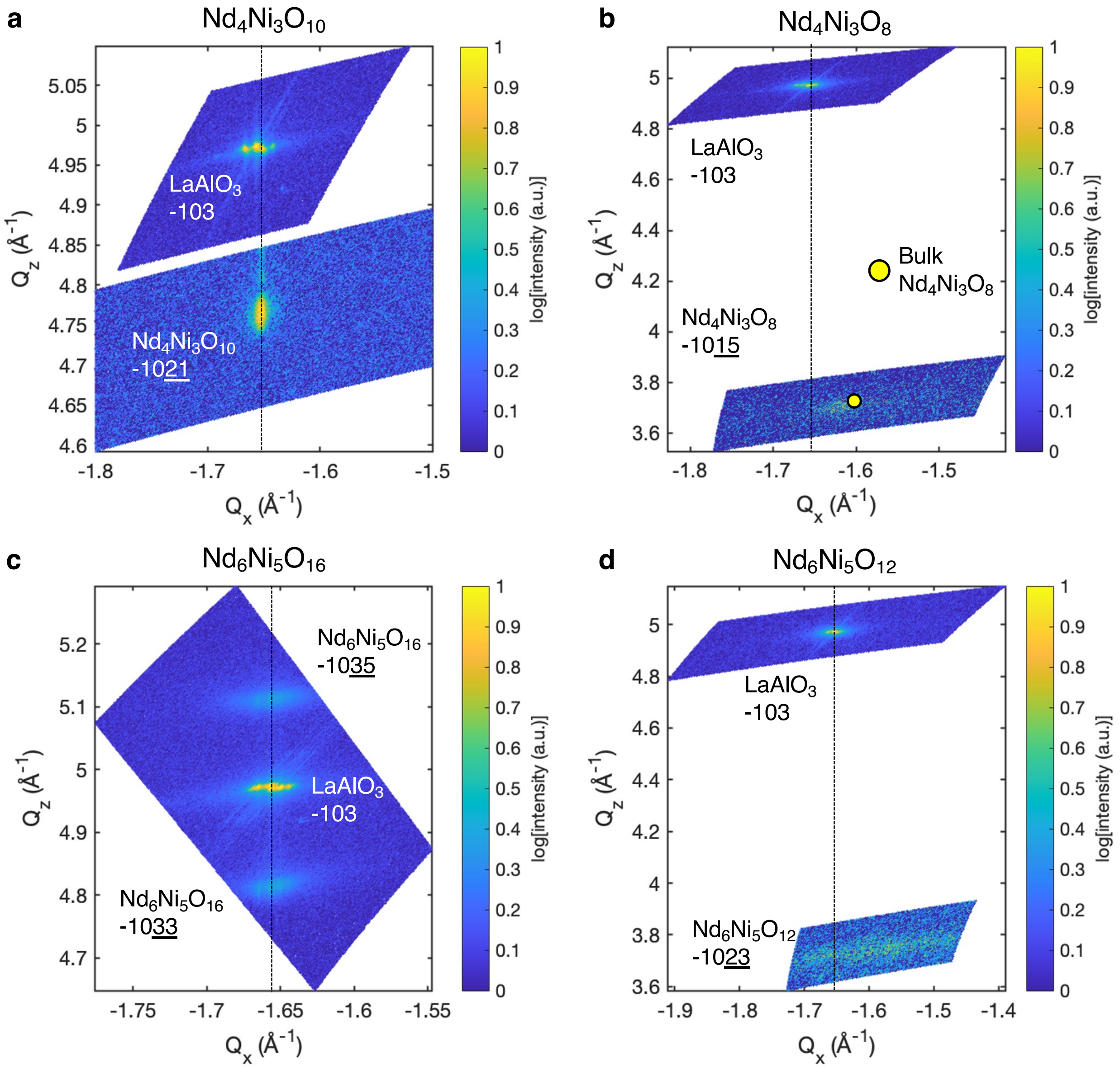

FIG. S12. Reciprocal space maps of (a) $Nd_4Ni_3O_{10}$ / $LaAlO_3$, (b) $Nd_4Ni_3O_8$ / $LaAlO_3$, (c) $Nd_6Ni_5O_{16}$ / $LaAlO_3$, and (d) $Nd_6Ni_5O_{12}$ / $LaAlO_3$. The reduced $n = 3$ film (20.8 thickness) is almost fully relaxed, with a 3.89 Å in-plane lattice parameter, compared to the 3.915 Åbulk value. The -10$\underline{15}$ peak of bulk $Nd_4Ni_3O_8$ is indicated by the yellow circle. The reduced $Nd_6Ni_5O_{12}$ film (43.1 nm) appears to be fully relaxed, with a 3.915 Å in-plane lattice parameter. The $n = 5$ peak is, however, very broad, likely due to the large thickness.

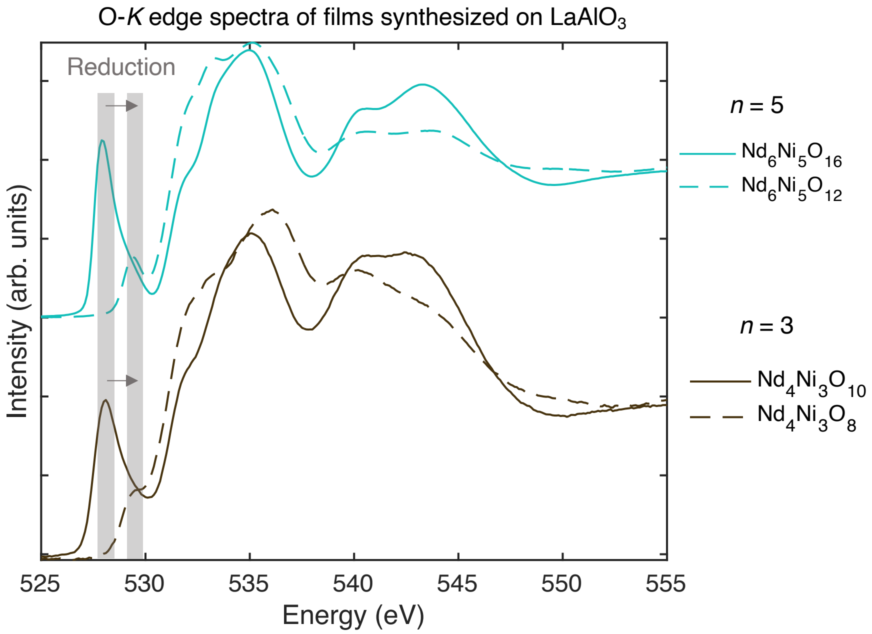

FIG. S13. Oxygen K-edge spectra of $n = 3$ and $n = 5$ films on $LaAlO_3$ before and after CaH_2 reduction. X-ray and transport characterization for these samples are provided in Figs. S6 and S9 with matching line colors. Upon reduction, the intense pre-peak shifts to higher energy and decreases in spectral weight. This shift is associated with a change in the oxygen - nickel hybridization from $2p$ - $3d^{7+\delta}$ to $2p$ - $3d^{9-\delta}$ [6]. A similar shift in the oxygen pre-peak has been observed in reduced layered nickelate films on $NdGaO_3$ [7], infinite-layer nickelates [8], and bulk compounds [9, 10].

SUPPLEMENTARY NOTE 5: REDUCTIONS ON $NdGaO_3$

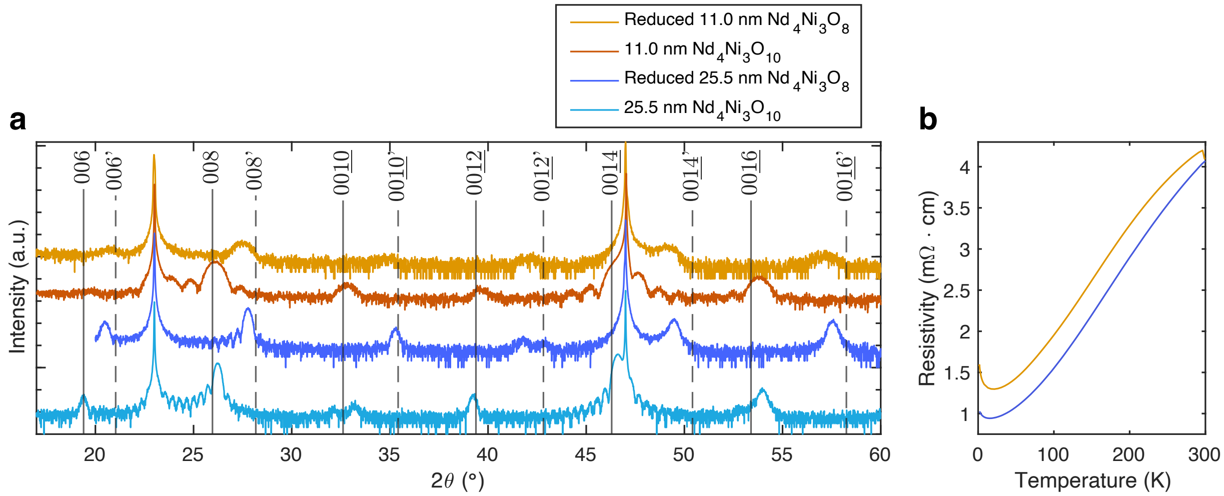

FIG. S14. Thickness-dependent reductions of $Nd_4Ni_3O_{10}$ / $NdGaO_3$. (a) XRD scans of as-grown and reduced films of 25.5 nm and 11.0 nm thicknesses. The 25.5 nm and 11.0 films were reduced for 3 hours at 300°C and 3 hours at 290°C, respectively. (b) Resistivity versus temperature measurements of the reduced films in (a).

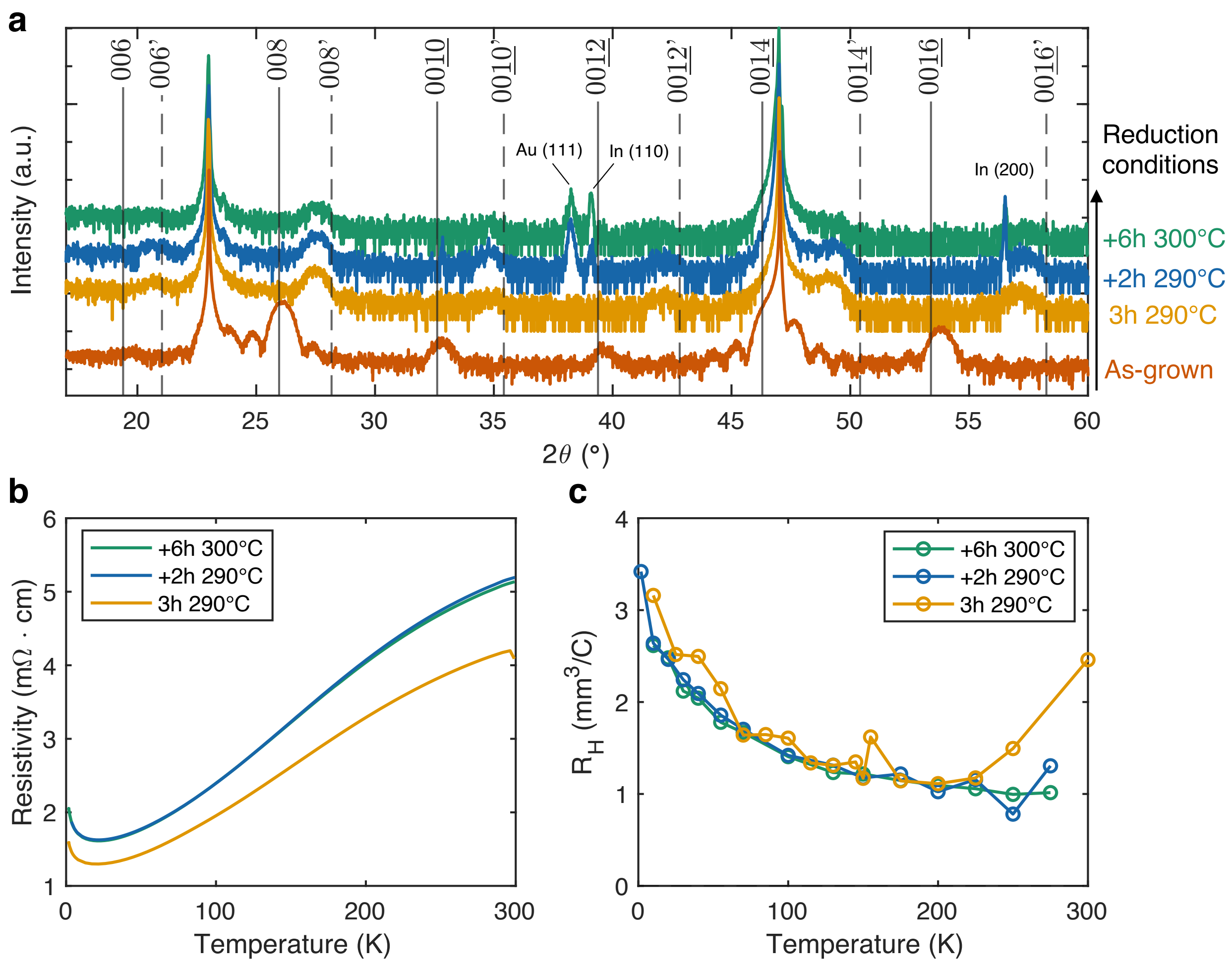

FIG. S15. X-ray and transport characterization of an incrementally reduced $Nd_4Ni_3O_{10}$ / $NdGaO_3$ film. (a) XRD scans of an as-grown film (red) then consecutively reduced for 3 hours at 290°C (yellow), 2 hours at 290°C (blue), and 6 hours at 300°C (green). (b) Resistivity versus temperature and (c) hall measurements of the reduced samples in (a).

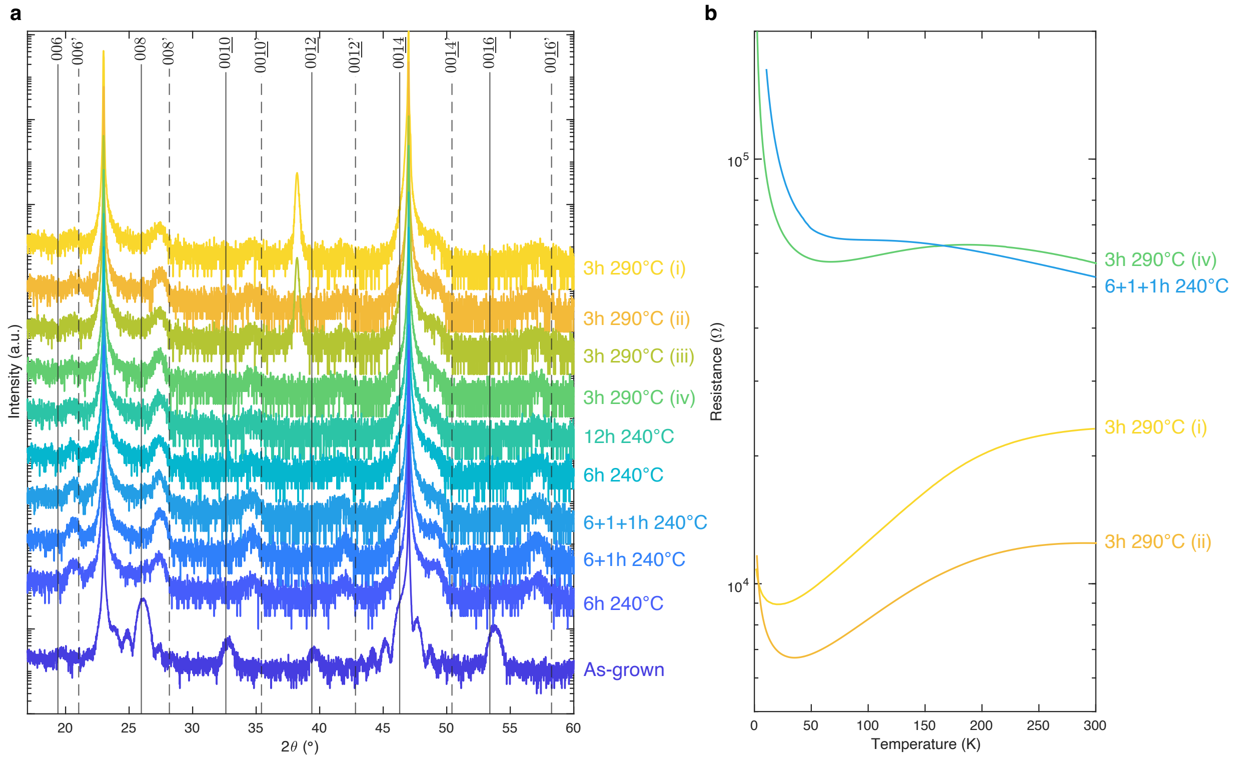

FIG. S16. Reductions of the 'optimal' $Nd_4Ni_3O_{10}$ / $NdGaO_3$ sample in main text Fig. 10 and Supplementary Note 8. (a) XRD scans of the as-grown $Nd_4Ni_3O_{10}$ and reduced $Nd_4Ni_3O_8$ films. Each scan is labeled with the reduction condition in hours and degrees Celsius. The peak at ∼37° corresponds to Au (111). (b) Resistance measurements of the films in (a).

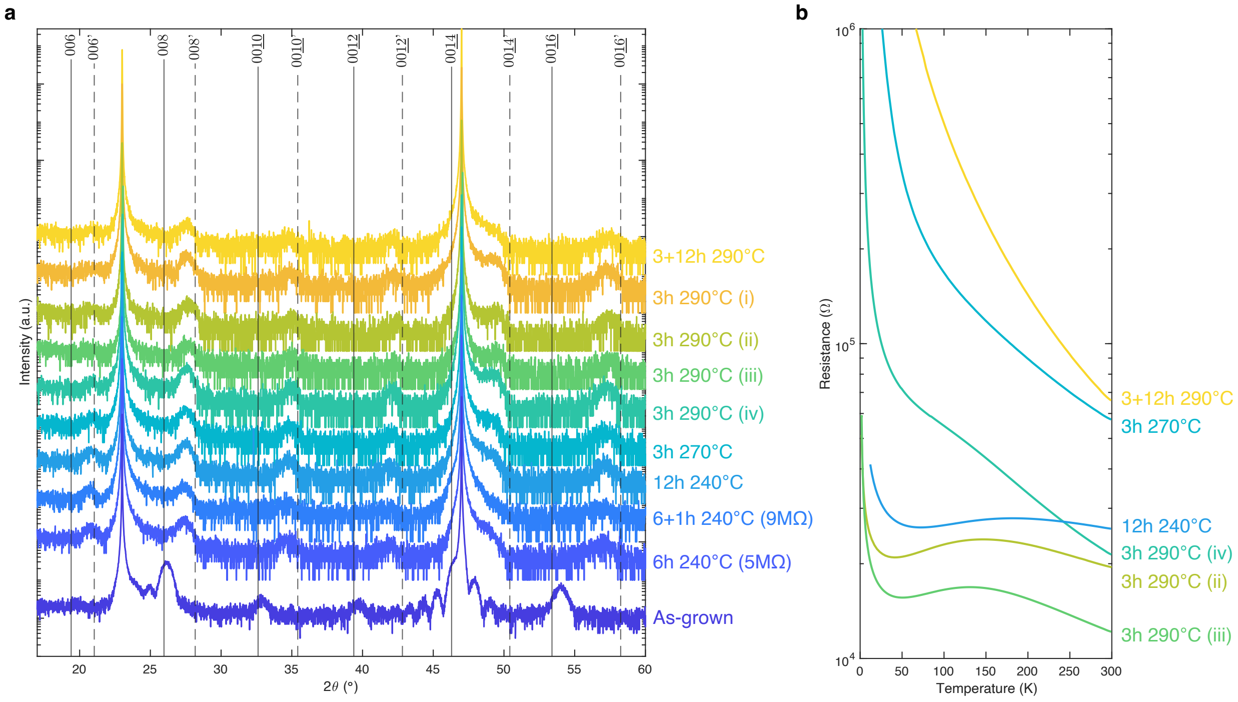

FIG. S17. Reductions of the 3% neodymium-poor $Nd_4Ni_3O_{10}$ / $NdGaO_3$ sample in main text Fig. 10 and Supplementary Note 8. (a) XRD scans of the as-grown $Nd_4Ni_3O_{10}$ and reduced $Nd_4Ni_3O_8$ films. Each scan is labeled with the reduction condition in hours and degrees Celsius. Some scans are also labeled with the surface resistance in parenthesis. The peak at ∼37° corresponds to Au (111). (b) Resistance measurements of the films in (a).

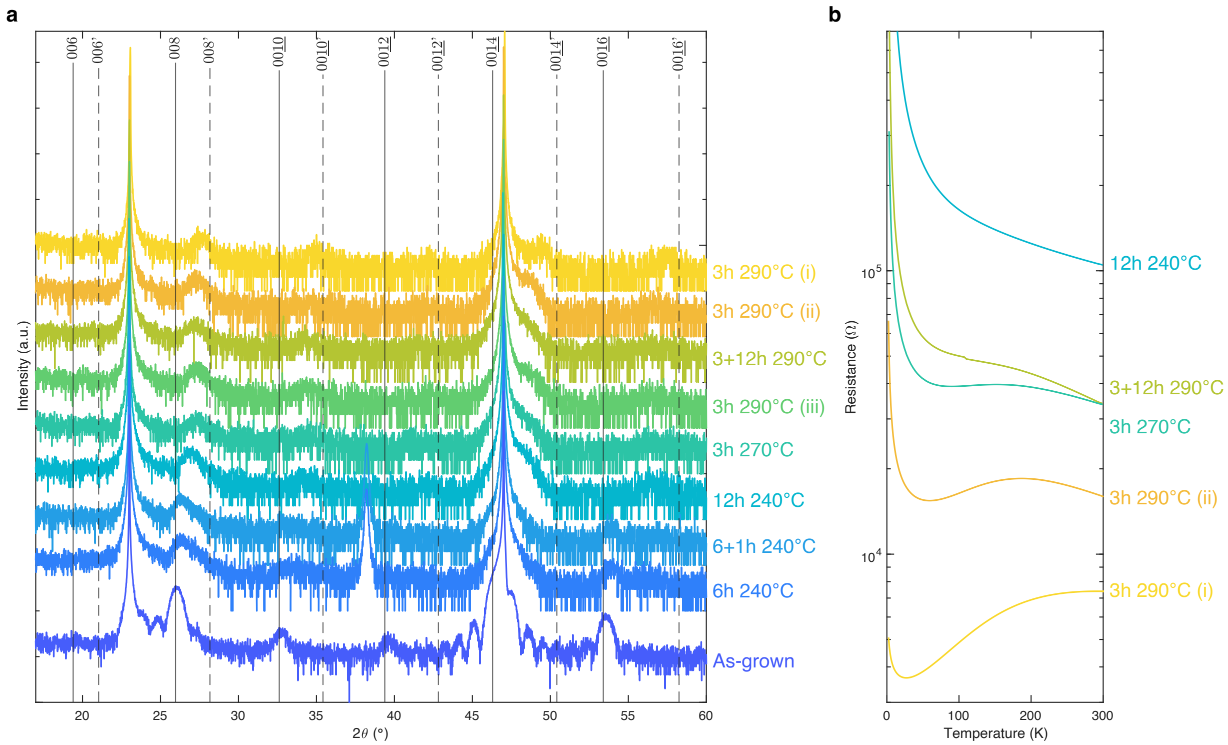

FIG. S18. Reductions of the 3% neodymium-rich $Nd_4Ni_3O_{10}$ / $NdGaO_3$ sample in main text Fig. 10 and Supplementary Note 8. (a) XRD scans of the as-grown $Nd_4Ni_3O_{10}$ and reduced $Nd_4Ni_3O_8$ films. Each scan is labeled with the reduction condition in hours and degrees Celsius. The peak at ∼37° corresponds to Au (111). (b) Resistance measurements of the films in (a).

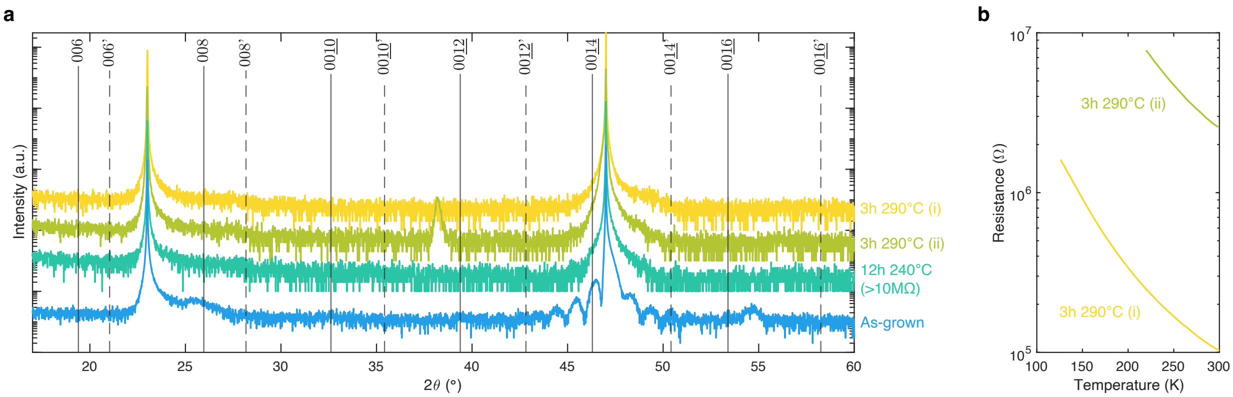

FIG. S19. Reductions of the 6% neodymium-poor $Nd_4Ni_3O_{10}$ / $NdGaO_3$ sample in main text Fig. 10 and Supplementary Note 8. (a) XRD scans of the as-grown $Nd_4Ni_3O_{10}$ and reduced $Nd_4Ni_3O_8$ films. Each scan is labeled with the reduction condition in hours and degrees Celsius. The sample reduced for 12 hours at 240°C is also labeled with the surface resistance in parenthesis. The peak at ∼37° corresponds to Au (111). (b) Resistance measurements of the films in (a).

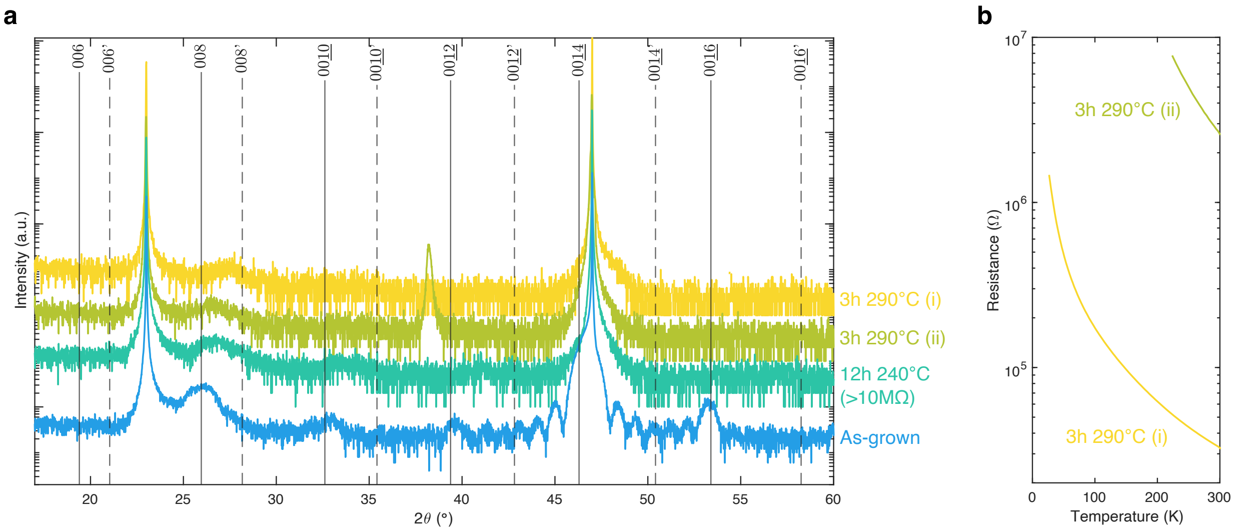

FIG. S20. Reductions of the 6% neodymium-rich $Nd_4Ni_3O_{10}$ / $NdGaO_3$ sample in main text Fig. 10 and Supplementary Note 8. (a) XRD scans of the as-grown $Nd_4Ni_3O_{10}$ and reduced $Nd_4Ni_3O_8$ films. Each scan is labeled with the reduction condition in hours and degrees Celsius. The sample reduced for 12 hours at 240°C is also labeled with the surface resistance in parenthesis. The peak at ∼37° corresponds to Au (111). (b) Resistance measurements of the films in (a).

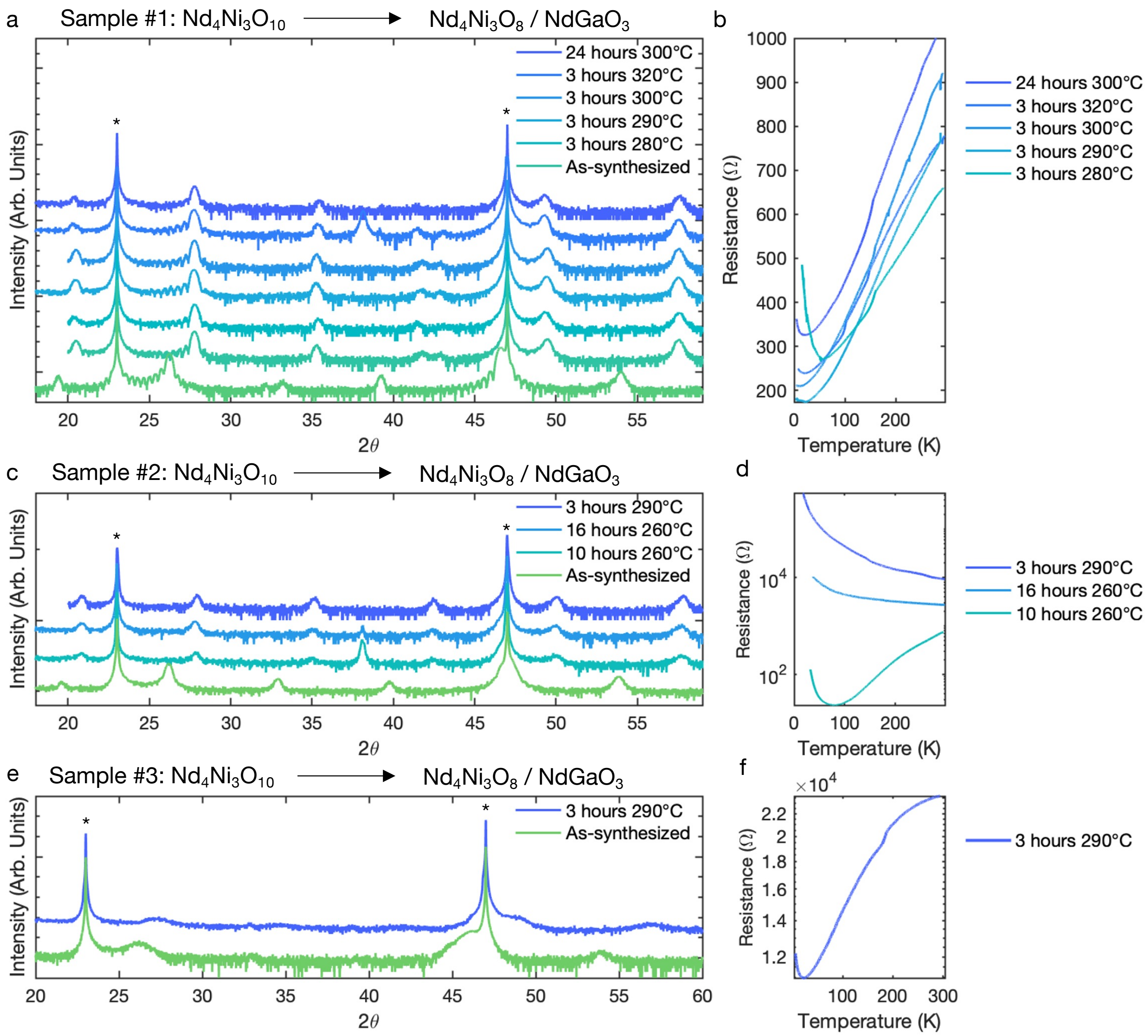

FIG. S21. XRD and resistance versus temperature measurements of three $Nd_4Ni_3O_{10}$ / $NdGaO_3$ films upon reduction: (a-b) sample #1, (c-d) sample #2, and (e-f) sample #3. The peak at $\sim 37^\circ$ in (a) and (b) is the gold 111 peak from electrical contacts. We note that sample #1 is metallic regardless of the large range in reduction conditions; the metallicity in sample #2 is sensitive to the reduction conditions; and sample #3 is metallic upon reduction, even though the structural quality by x-ray is inferior to that of both samples #1 or #2. Resistance measurements were taken using a home-built 'dipstick probe'.

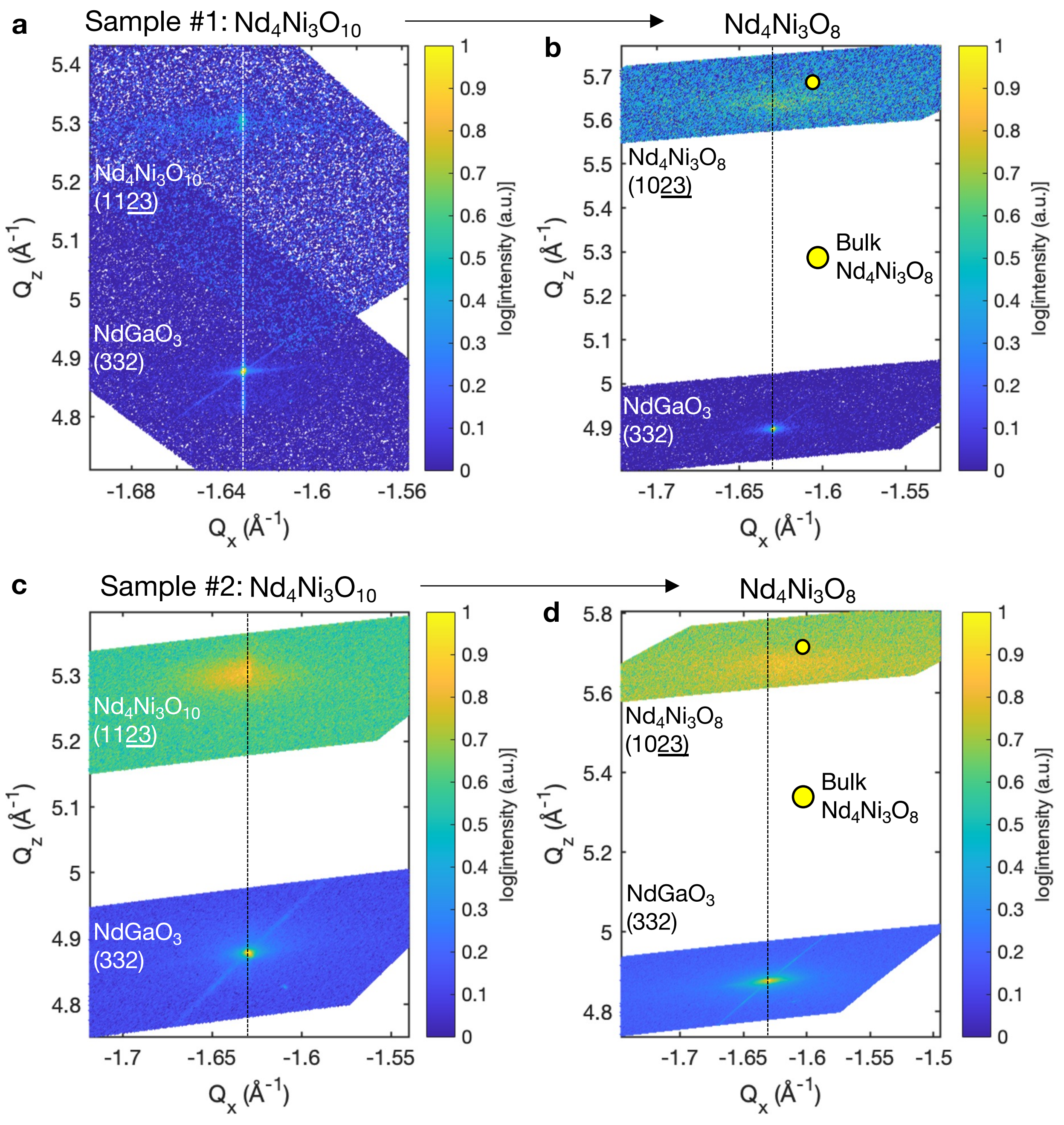

FIG. S22. Reciprocal space maps of (a,c) $Nd_4Ni_3O_{10}$ and (b,d) $Nd_4Ni_3O_8$ films on $NdGaO_3$ (110). (a-b) Sample #1 (Figs. 8 and S21(a-b)) and (c-d) sample #2 (Fig. S21(c-d)). Samples #1 and #2 were reduced for 3 hours at 290°C and 10 hours at 260°C, respectively.

SUPPLEMENTARY NOTE 6: ATOMIC STRUCTURE OF THE FILM-SUBSTRATE INTERFACES

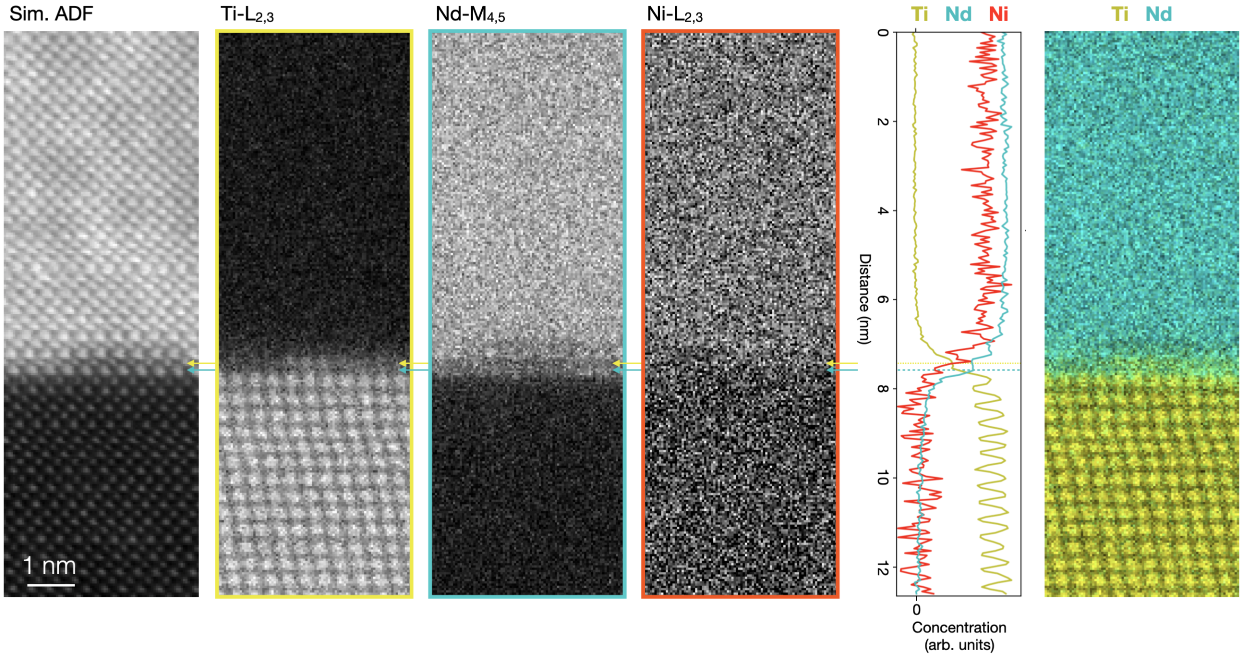

FIG. S23. Simultaneously acquired annular dark-field (Sim. ADF) image and atomic-resolution electron energy loss spectroscopy (EELS) elemental maps of the interface between the $Nd_4Ni_3O_{10}$ film and $SrTiO_3$ substrate shows a similar single unit cell intermediate layer of $\sim$Nd(Ti,Ni)O_3 as that observed and characterized for infinite-layer films on $SrTiO_3$ [11]. Spectroscopic studies and theoretical calculations of the intermediate layer in perovskite/infinite-layer thin films show that it alleviates the strong polar discontinuity which would otherwise form at an abrupt interface between the charge neutral planes of $SrTiO_3$ and the charged planes in the nickelate film. The atomic contrasts of Nd and Ni are washed out in most of the film due to the significant concentration of Ruddelsden-Popper faults. Summed concentration profiles for the Ti-$L_{2,3}$, Nd-$M_{4,5}$, and Ni-$L_{2,3}$ maps are also shown, along with a false color overlay of the Ti-$L_{2,3}$ (yellow) and Nd-$M_{4,5}$ (cyan) maps. Yellow and cyan arrows and dashed lines mark the atomic planes of the intermediate layer. The apparent skew of the ADF image and EELS maps is from sample drift during the EELS acquisition.

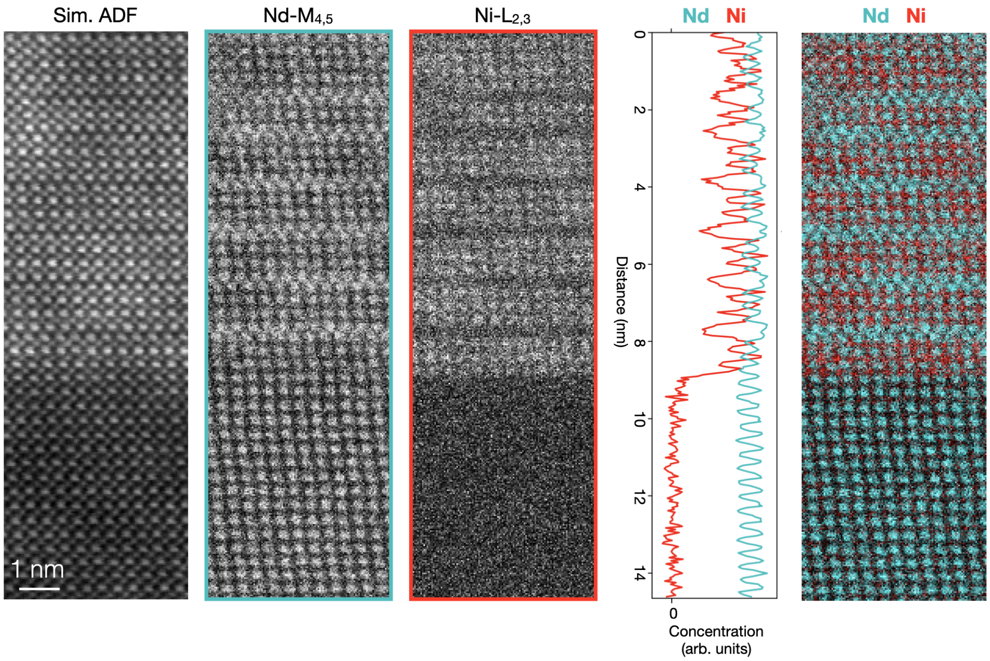

FIG. S24. Simultaneously acquired annular dark-field (Sim. ADF) image and atomic-resolution electron energy loss spectroscopy (EELS) elemental maps of the interface between the $Nd_4Ni_3O_{10}$ film and $NdGaO_3$ substrate. The formal planar charge alterations of $NdGaO_3$ are $\pm$ 1, close to the formal planar charges in the nickelate film. Summed concentration profiles for the Nd-$M_{4,5}$ and Ni-$L_{2,3}$ maps are also shown, along with a false color overlay of the Nd-$M_{4,5}$ (cyan) and Ni-$L_{2,3}$ (red) maps. The apparent skew of the ADF image and EELS maps is from sample drift during the EELS acquisition.

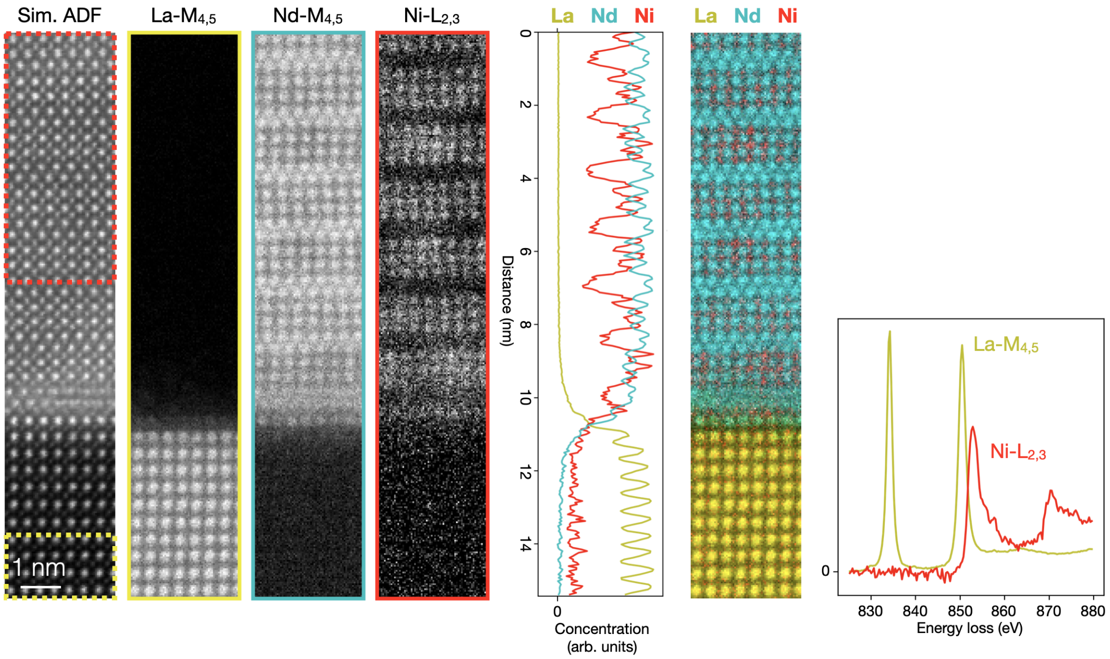

FIG. S25. Simultaneously acquired annular dark-field (Sim. ADF) image and atomic-resolution electron energy loss spectroscopy (EELS) elemental maps of the interface between the $Nd_4Ni_3O_8$ film and $LaAlO_3$ substrate. The formal planar charge alterations of $LaAlO_3$ are $\pm$ 1, close to the formal planar charges in the nickelate film. The half-unit of $Nd_4Ni_3O_8$ (i.e., single NiO_2 plane below the first fluorite layer) is due to a different shuttering sequence used in the growth of this film as compared to the full $Nd_4Ni_3O_{10}$ unit (three NiO_2 planes before the first rock salt plane) observed in the film grown on $NdGaO_3$ shown in Figure S24. Due to their overlapping EELS edges, two-dimensional concentration maps of the La-$M_{4,5}$ and Ni-$L_{2,3}$ edges are determined by non-negative least squares (NNLS) fit to the weighted sum of reference components for each edge taken from substrate (La-$M_{4,5}$) and film (Ni-$L_{2,3}$) regions marked by the yellow and red dashed boxes on the simultaneous ADF. Summed concentration profiles for the La-$M_{4,5}$, Nd-$M_{4,5}$, and Ni-$L_{2,3}$ maps are also shown, along with a false color overlay of the La-$M_{4,5}$ (yellow), Nd-$M_{4,5}$ (cyan), and Ni-$L_{2,3}$ (red) maps. The reference components for the La-$M_{4,5}$ and Ni-$L_{2,3}$ concentration mapping are shown at the far right.

SUPPLEMENTARY NOTE 7: STRUCTURAL CHARACTERIZATION BY SCANNING TRANSMISSION ELECTRON MICROSCOPY

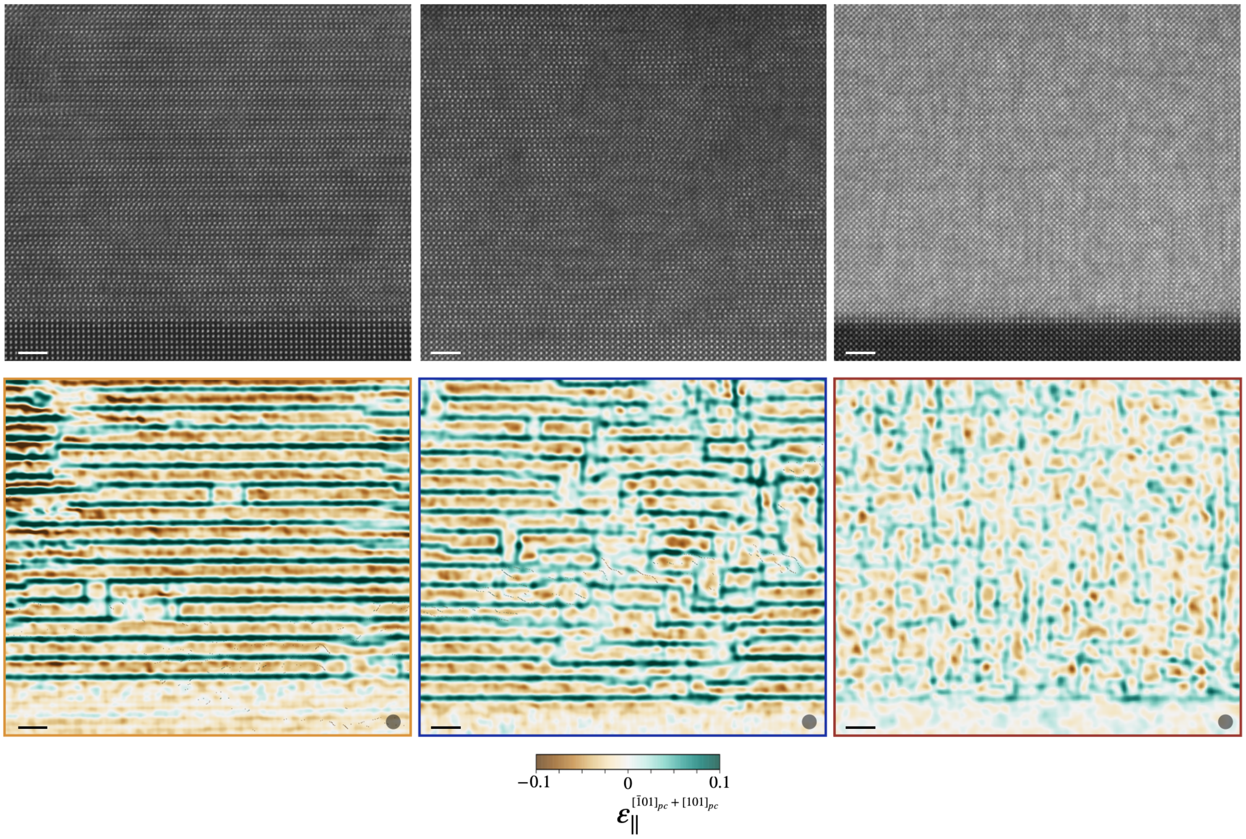

FIG. S26. Raw HAADF-STEM images and measured tensile strain in the [$\bar{1}$01] and [101] pseudocubic lattice fringes for as-synthesized $Nd_4Ni_3O_{10}$ films on $LaAlO_3$ (left), $NdGaO_3$ (center), and $SrTiO_3$ (right) shown in Figure 5 of the main text. "Strain" refers to the local expansion or contraction of the lattice fringes from a reference vector rather than real elastic lattice strain. Maps are generated using phase lock-in analysis described in Goodge *et al.* [12]. Scale bars are 2 nm. Black circles denote the coarsening length scale of the Fourier-based phase analysis.

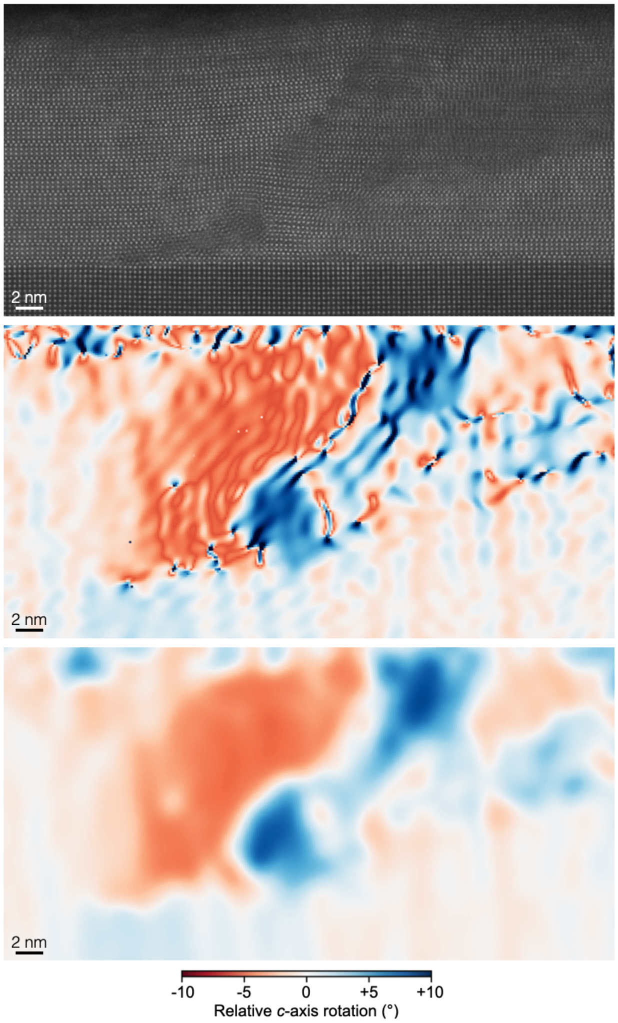

FIG. S27. Raw HAADF-STEM image (top) with raw (middle) and Gaussian-smoothed (bottom) relative c-axis rotation maps corresponding to Figure 7(e) of the main text. The c-axis rotation is measured by the local wavefitting procedure described in Smeaton *et al.* [13] and is displayed relative to the average orientation within the full image. Dislocations in the lattice rotation map appear at lattice slips or vertical Ruddlesden-Popper faults within the film lattice. The overlaid orientation map in Figure 7(e) is smoothed by a Gaussian kernel with $\sigma = 5$, corresponding to a distance of about 5 pseudocubic unit cells.

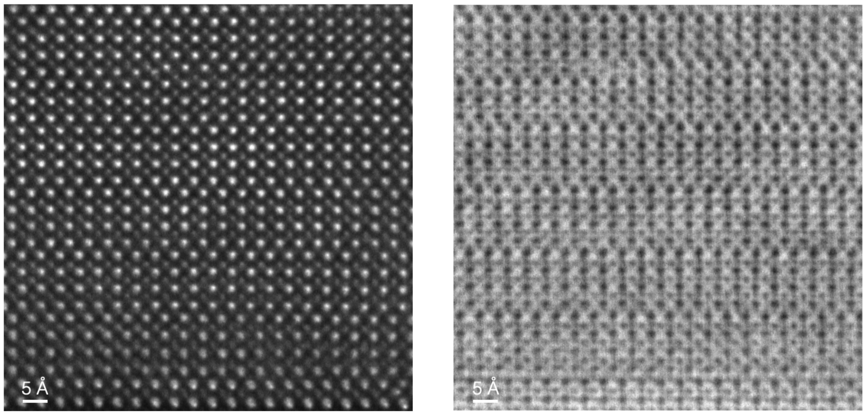

FIG. S28. Full field-of-view HAADF- (left) and ABF- (right) STEM images of the reduced $Nd_4Ni_3O_8$ film on $LaAlO_3$ from which the regions shown in Figure 7(c) and (d) are taken.

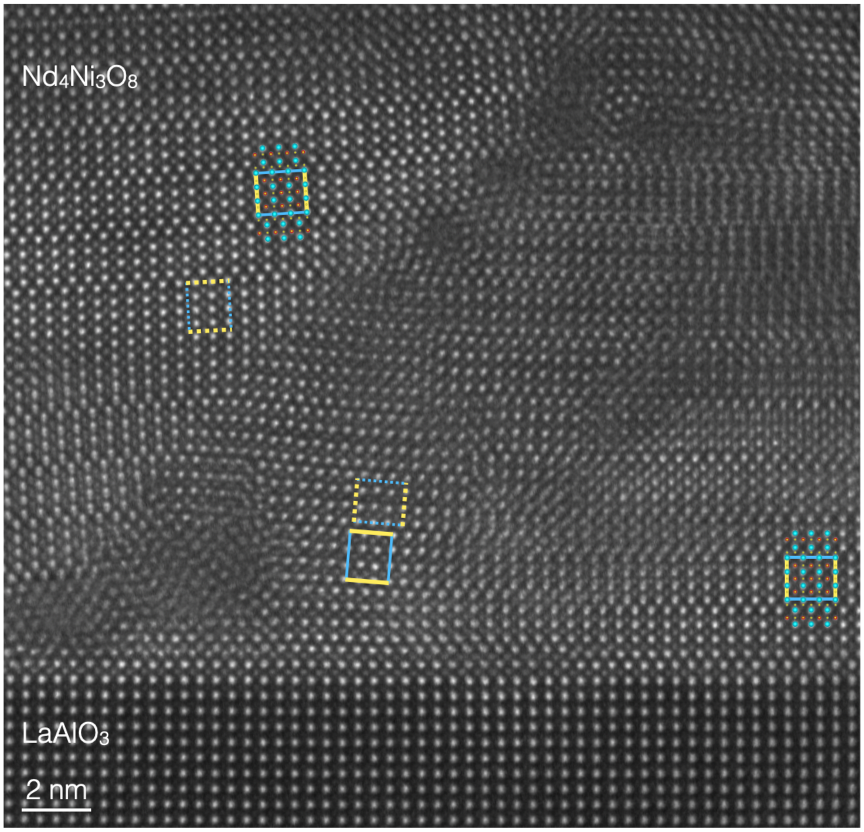

FIG. S29. High-resolution HAADF-STEM image from the same diagonal defect shown in Figure 7(e) of the main text with local lattice symmetries marked by cyan and yellow rectangles. Yellow and cyan sides correspond to short and long axes, respectively, of each rectangle. Dashed boxes show comparisons for 90°-rotated rectangles near the defect which clearly do not match the observed lattice at each location. While the c-axis orientation of the full Ruddlesden-Popper structure is preserved globally (other than the few-degree tilting shown in Figures 7(e) and S27), the pseudo-infinite-layer structure within each Ruddlesden-Popper layer shows some variation near the diagonal defect. Far from the defect (e.g., near the right edge of the image), the layered short axis (yellow) is aligned with Ruddlesden-Popper c-axis (i.e., out-of-plane) and the layered long axis (cyan) is in the plane. To the left of the diagonal defect, the same structure is observed with a few-degree rotation of the lattice, as discussed in Figures 7(e) and S27. To the right of the diagonal defect, however, the $n = 3$ Ruddlesden-Popper structure is retained, but the short (yellow) axis falls in-plane while the longer (cyan) axis is out-of-plane. This change in local lattice symmetry suggests local regions with an intra-Ruddlesden-Popper rotation of the layered structure – possibly similar to that reported in reduced $LaNiO_2$ films on $SrTiO_3$ [14] – with the Nd and NiO_2 planes oriented orthogonal to the substrate.

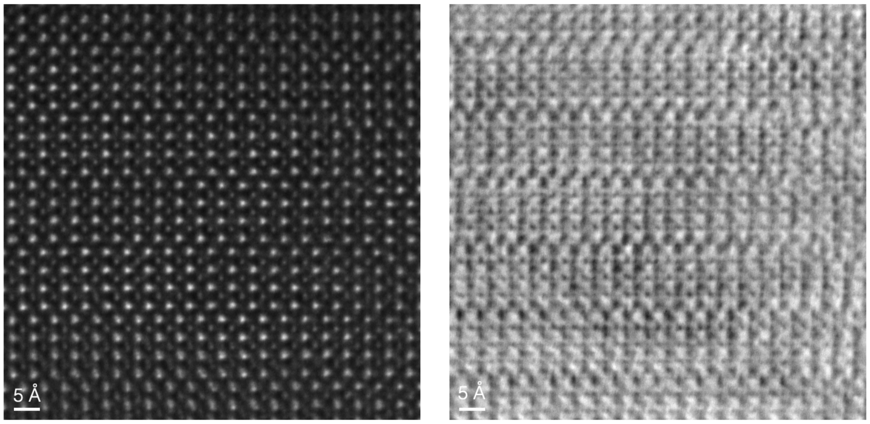

FIG. S30. Full field-of-view HAADF- (left) and ABF- (right) STEM images of the reduced $Nd_4Ni_3O_8$ film on $NdGaO_3$ from which the regions shown in Figure 9(e) and (f) are taken.

SUPPLEMENTARY NOTE 8: CATION STOICHIOMETRY

To investigate the role of cation stoichiometry (Nd/Ni ratio), we synthesized a series of five $Nd_4Ni_3O_{10}$ / $NdGaO_3$ films with varying neodymium content, which we will refer to as the 'stoichiometry series'. We synthesized the series within twenty four hours to ensure chamber stability. We verify the chamber stability by re-synthesizing the first film in the series and verifying that the structure by XRD is nearly identical. We vary the neodymium content by 3% between each sample by altering the neodymium monolayer shutter time while keeping the nickel shutter time and all other growth parameters constant. Due to the difficulty in precisely measuring the neodymium content in neodymium nickelate films on $NdGaO_3$ by RBS, we do not assign compositions to the samples and instead refer to the percentage of neodymium relative to the optimal sample, which exhibits the highest intensity XRD peaks (Fig. S31).

In Fig. S31 we present the structural characterization of the $Nd_4Ni_3O_{10}$ / $NdGaO_3$ stoichiometry series. The optimal sample in Fig. S31(a) exhibits the highest intensity superlattice peaks of all samples in the series and is the only sample with a clear 006 peak. The most off-stoichiometric samples (-6% and +6% neodymium) exhibit much lower intensity XRD peaks; notably, the neodymium-deficient (-6%) sample possesses a considerably inferior structure to the neodymium-rich (+6%) film. The systematic left-ward shift in the 0016 peak location with increasing neodymium content highlighted in Fig. S31(b) is indicative of an expansion in the c-axis lattice constant, shown in Fig. S31(c). The contracted c-axis lattice constants with respect to the bulk $Nd_4Ni_3O_{10}$ value is a consequence of the tensile strain (see Fig. S5). The observed expansion in the c-axis lattice constant with neodymium-richness is consistent with neodymium-rich $NdNiO_3$ films [15, 16].

Fig. S31 displays the structural characterization of the reduced $Nd_4Ni_3O_8$ stoichiometry series. The three samples within 3% of the optimal neodymium content in Fig. S31(d) exhibit all expected XRD peaks while the off-stoichiometric ±6% show very few peaks. Fig. S31(e) show hints of a systematic trend in 008 peak position with neodymium content, but the large error in the c-axis lattice parameter estimates shown in Fig. S31(f) preclude the determination of a trend similar to the one evident in the as-grown samples in Fig. S31(c). The expanded c-axis lattice constants are consistent with the nominal compressive strain in the reduced state.

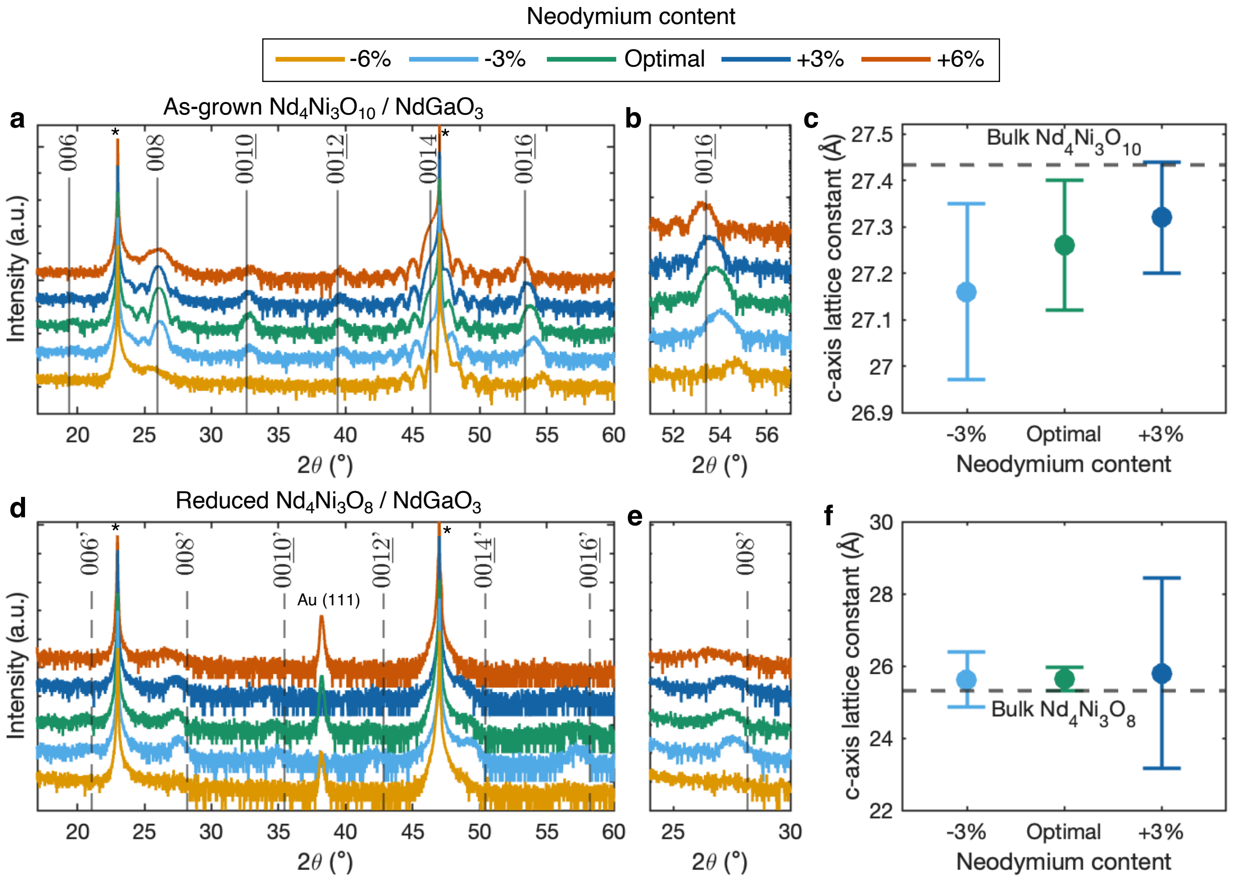

FIG. S31. Structural characterization of (a-c) $Nd_4Ni_3O_{10}$ and (d-f) $Nd_4Ni_3O_8$ films on $NdGaO_3$ (110) with varying neodymium content. All films are reduced for 3 hours at 290°C. (a) XRD scans of $Nd_4Ni_3O_{10}$ / $NdGaO_3$ films with varying neodymium content (controlled by shutter times in the MBE) within 6% of the optimal value. (b) Zoom-in of the 0016 peaks. (c) *c*-axis lattice constants calculated by Nelson-Riley fits of the superlattice peaks in (a). (d) XRD scans of $Nd_4Ni_3O_8$ / $NdGaO_3$ films reduced from those shown in (a). (e) Zoom-in of the 008 peaks. (f) *c*-axis lattice constants calculated by Nelson-Riley fits of the superlattice peaks in (d).

Next we investigate how off-stoichiometry influences the electronic properties of $Nd_4Ni_3O_{10}$. Via formal electron counting, we can estimate that $Nd_{4+x}Ni_3O_{10}$ will possess a $+2.67 - x$ nickel valence and $3d^{7.33+x}$ electron count. Therefore, excess neodymium may effectively electron dope the system. Measurements of the hall coefficent in Fig. S32(a) suggest a trend in carrier density with neodymium content at low temperature, shown in Fig. S32(b). Taking the hall coefficients measured at 10K, the carrier density decreases with increasing neodymium content (Fig. S32(c)).

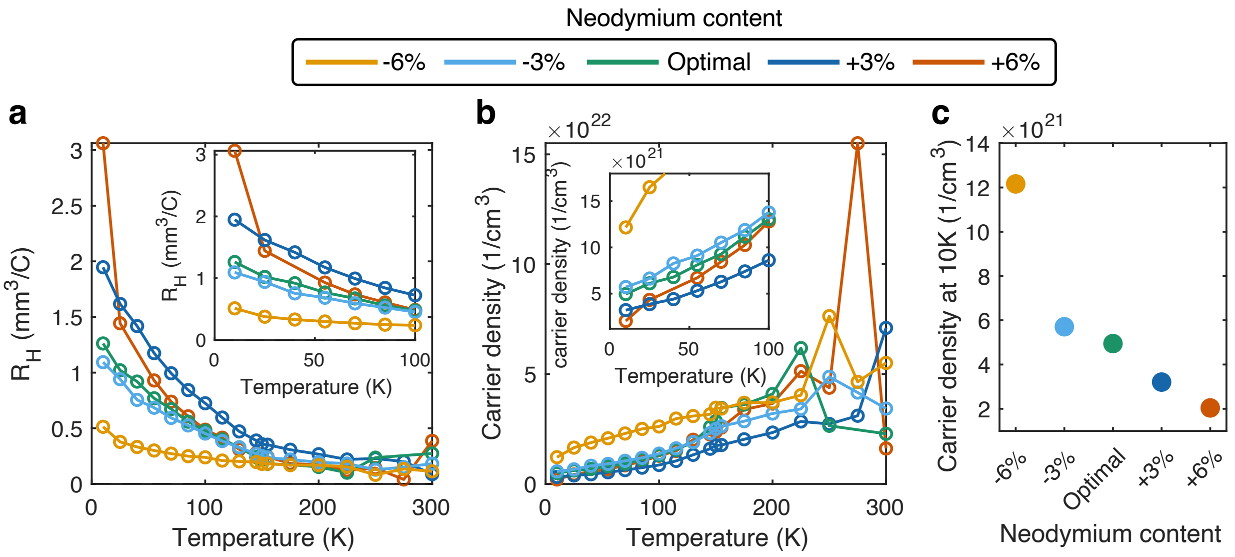

FIG. S32. (a) Hall coefficient versus temperature measurements of $Nd_4Ni_3O_{10}$ / $NdGaO_3$ (110) films with varying neodymium content. (b) Carrier density versus temperature and (c) carrier density at 10K versus neodymium content.

We can also verify changes to the nickel filling in the $Nd_4Ni_3O_{10}$ using x-ray absorption spectroscopy (XAS). In Fig. S33(a), we note the presence of two multiplet peaks at the nickel L_3 edge, labelled A and B. As we increase the neodymium content, we see a consistent diminishing of the peak B intensity relative to the peak A intensity. The relative increase of the A/B intensity ratio has been associated with a shift in nickel valence (filling) from 3+ (d^7) to 2+ (d^8) irrespective of the method of charge transfer, including: oxygen vacancy formation [17–19], modulation of Ruddlesden-Popper order [20], and interfacial doping [21]. The overall width of the nickel L_3 edge also decreases with increasing neodymium, further reflecting a decrease in intensity of the B peak, which is the broader of the two multiplet features. Here then, the XAS clearly suggest that the formal nickel valence (filling) decreases (increases) with increasing neodymium content.

However, after reducing all five samples to the $Nd_4Ni_3O_8$ phase, we see no clear trends across the nickel L_3 edge (Fig. S33(b)). Assuming a perfectly oxygen-stoichiometric phase, if neodymium off-stoichiometry could effectively dope the nickel site, we would expect the relative broadening of the nickel L_3 edge to increase with increasing neodymium [8, 22]. We do not see this behavior. This indicates that the reduction process introduces an inherent randomness that potentially targets the oxygen stoichiometry, which may wash out obvious trends from cationic stoichiometry in spectroscopic probes. Nonetheless, the resistivity variation across the $Nd_4Ni_3O_8$ (Fig. 10(f)) still suggests that the stoichiometric composition of the as-synthesized $Nd_4Ni_3O_{10}$ phase is paramount in achieving metallic behavior in the reduced $Nd_4Ni_3O_8$ phase.

We note that in the octahedrally-coordinated nickelates, a change in nickel valence from

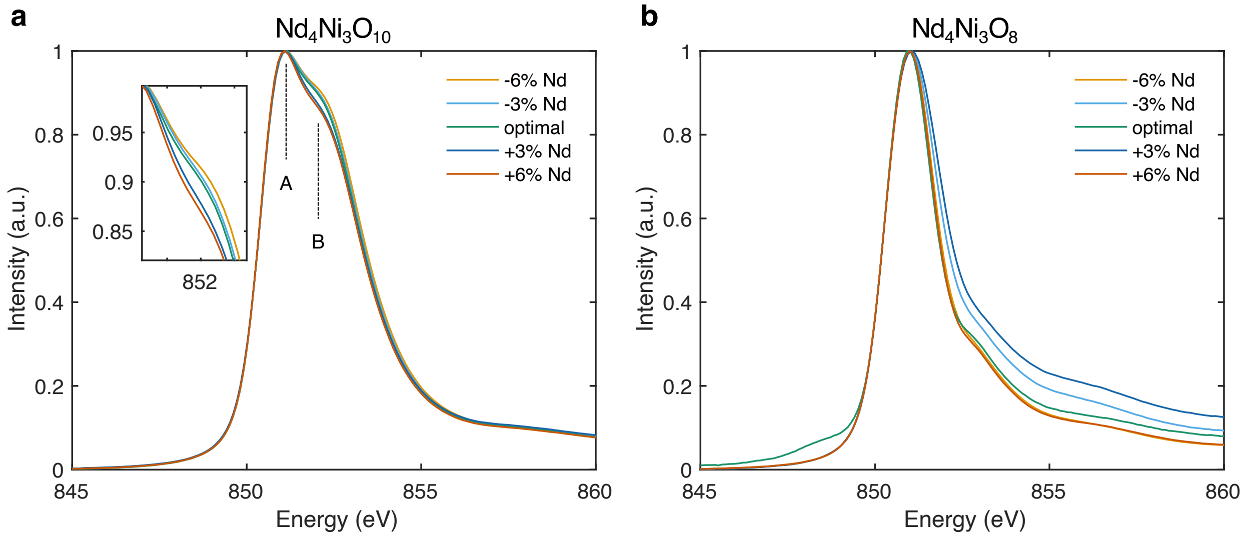

FIG. S33. Nickel L_3 edge x-ray absorption spectra of the (a) as-synthesized $Nd_4Ni_3O_{10}$ and (b) reduced $Nd_4Ni_3O_8$ phases for a series of films with varying neodymium content. The inset in (a) is a zoom-in of the 'B' multiplet peak to highlight the decrease in B peak intensity with increasing neodymium content.

3+ to 2+ should also be accompanied by a very gradual shifting of peak A to lower energy [17]. However, due to the incident energy variation at the endstation, which can be up to 0.4 eV, we cannot resolve this shift confidently. As a result, we do not attempt to extract this potential trend, and have artificially shifted the spectra so that the onset of the nickel L_3 edges align to the average onset of the five spectra, for both the $Nd_4Ni_3O_{10}$ and $Nd_4Ni_3O_8$ cases. This enables a clearer visualization of relative peak intensities and widths. We have also scaled the data to the intensity at the pre-edge, and performed a min-max normalization over the entire nickel L_3 edge.

[1] G. Kresse and J. Furthmüller, Efficient iterative schemes for ab initio total-energy calculations using a plane-wave basis set, Phys. Rev. B **54**, 11169 (1996).

[2] J. P. Perdew, K. Burke, and M. Ernzerhof, Generalized gradient approximation made simple, Phys. Rev. Lett. **77**, 3865 (1996).

[3] M. Kitatani, L. Si, O. Janson, R. Arita, Z. Zhong, and K. Held, Nickelate superconductors—a renaissance of the one-band hubbard model, npj Quantum Materials **5**, 59 (2020).

[4] H. LaBollita, M.-C. Jung, and A. S. Botana, Many-body electronic structure of $d^{9-\delta}$ layered nickelates, Phys. Rev. B **106**, 115132 (2022).

[5] A. Olafsen, H. Fjellvåg, and B. C. Hauback, Crystal Structure and Properties of $Nd_4Co_3O_{10+\delta}$ and $Nd_4Ni_3O_{10-\delta}$, Journal of Solid State Chemistry **151**, 46 (2000).

[6] M. Abbate, G. Zampieri, F. Prado, A. Caneiro, J. M. Gonzalez-Calbet, and M. Vallet-Regi, Electronic structure and metal-insulator transition in $LaNiO_{3-\delta}$, Physical Review B **65**, 155101 (2001).

[7] G. A. Pan, D. F. Segedin, H. LaBollita, Q. Song, E. M. Nica, B. H. Goodge, A. T. Pierce, S. Doyle, S. Novakov, D. C. Carrizales, A. T. N'Diaye, P. Shafer, H. Paik, J. T. Heron, J. A. Mason, A. Yacoby, L. F. Kourkoutis, O. Erten, C. M. Brooks, A. S. Botana, and J. A. Mundy, Superconductivity in a quintuple-layer square-planar nickelate, Nature Materials **21**, 160 (2021).

[8] B. H. Goodge, D. Li, K. Lee, M. Osada, B. Y. Wang, G. A. Sawatzky, H. Y. Hwang, and L. F. Kourkoutis, Doping evolution of the Mott–Hubbard landscape in infinite-layer nickelates, Proceedings of the National Academy of Sciences **118**, e2007683118 (2021).

[9] J. Zhang, A. S. Botana, J. W. Freeland, D. Phelan, H. Zheng, V. Pardo, M. R. Norman, and J. F. Mitchell, Large orbital polarization in a metallic square-planar nickelate, Nature Physics **13**, 864 (2017).

[10] J. Q. Lin, P. V. Arribi, G. Fabbris, A. S. Botana, D. Meyers, H. Miao, Y. Shen, D. G. Mazzone, J. Feng, S. G. Chiuzbăian, A. Nag, A. C. Walters, M. García-Fernández, K.-J. Zhou, J. Pelliciari, I. Jarrige, J. W. Freeland, J. Zhang, J. F. Mitchell, V. Bisogni, X. Liu, M. R. Norman, and M. P. M. Dean, Strong Superexchange in a $d^{9-\delta}$ Nickelate Revealed by Resonant Inelastic X-Ray Scattering, Physical Review Letters **126**, 087001 (2021).

[11] B. H. Goodge, B. Geisler, K. Lee, M. Osada, B. Y. Wang, D. Li, H. Y. Hwang, R. Pentcheva, and L. F. Kourkoutis, Reconstructing the polar interface of infinite-layer nickelate thin films 10.48550/arxiv.2201.03613 (2022).

[12] B. H. Goodge, I. El Baggari, S. S. Hong, Z. Wang, D. G. Schlom, H. Y. Hwang, and L. F. Kourkoutis, Disentangling coexisting structural order through phase lock-in analysis of atomic-resolution STEM data, Microscopy and Microanalysis **28**, 404–411 (2022).

[13] M. A. Smeaton, I. E. Baggari, D. M. Balazs, T. Hanrath, and L. F. Kourkoutis, Mapping

Defect Relaxation in Quantum Dot Solids upon *In Situ* Heating, ACS Nano **15**, 719 (2021).

[14] M. Osada, B. Y. Wang, B. H. Goodge, S. P. Harvey, K. Lee, D. Li, L. F. Kourkoutis, and H. Y. Hwang, Nickelate Superconductivity without Rare-Earth Magnetism: (La,Sr)NiO_2, Advanced Materials **33**, 2104083 (2021).

[15] E. Breckenfeld, Z. Chen, A. R. Damodaran, and L. W. Martin, Effects of Nonequilibrium Growth, Nonstoichiometry, and Film Orientation on the Metal-to-Insulator Transition in $NdNiO_3$ Thin Films, ACS Applied Materials & Interfaces **6**, 22436 (2014).

[16] Y. Li, W. Sun, J. Yang, X. Cai, W. Guo, Z. Gu, Y. Zhu, and Y. Nie, Impact of Cation Stoichiometry on the Crystalline Structure and Superconductivity in Nickelates, Frontiers in Physics **9**, 719534 (2021).

[17] J. W. Freeland, M. Van Veenendaal, and J. Chakhalian, Evolution of electronic structure across the rare-earth rnio3 series, Journal of Electron Spectroscopy and Related Phenomena **208**, 56 (2016).

[18] I.-C. Tung, G. Luo, J. H. Lee, S. H. Chang, J. Moyer, H. Hong, M. J. Bedzyk, H. Zhou, D. Morgan, D. D. Fong, *et al.*, Polarity-driven oxygen vacancy formation in ultrathin $lanio_3$ films on $srtio_3$, Physical Review Materials **1**, 053404 (2017).

[19] T. Kim, T. Paudel, R. Green, K. Song, H.-S. Lee, S.-Y. Choi, J. Irwin, B. Noesges, L. Brillson, M. Rzchowski, *et al.*, Strain-driven disproportionation at a correlated oxide metal-insulator transition, Physical Review B **101**, 121105 (2020).

[20] G. A. Pan, Q. Song, D. F. Segedin, M.-C. Jung, H. El-Sherif, E. E. Fleck, B. H. Goodge, S. Doyle, D. C. Carrizales, A. T. N'Diaye, P. Shafer, H. Paik, L. F. Kourkoutis, I. E. Baggari, A. S. Botana, C. M. Brooks, and J. A. Mundy, Synthesis and electronic properties of $Nd_{n+1}Ni_nO_{3n+1}$ Ruddlesden-Popper nickelate thin films, Physical Review Materials **6**, 055003 (2022).

[21] A. S. Disa, D. P. Kumah, A. Malashevich, H. Chen, D. A. Arena, E. D. Specht, S. Ismail-Beigi, F. Walker, and C. H. Ahn, Orbital engineering in symmetry-breaking polar heterostructures, Physical Review Letters **114**, 026801 (2015).

[22] M. Rossi, H. Lu, A. Nag, D. Li, M. Osada, K. Lee, B. Y. Wang, S. Agrestini, M. Garcia-Fernandez, J. Kas, *et al.*, Orbital and spin character of doped carriers in infinite-layer nickelates, Physical Review B **104**, L220505 (2021).
