# Supplementary figures and images for: Limits to the strain engineering of layered square-planar nickelate thin films
Source: Nat Commun. 2023 Mar 16;14:1468. doi: 10.1038/s41467-023-37117-4 (PMC10020545; doi:10.1038/s41467-023-37117-4)

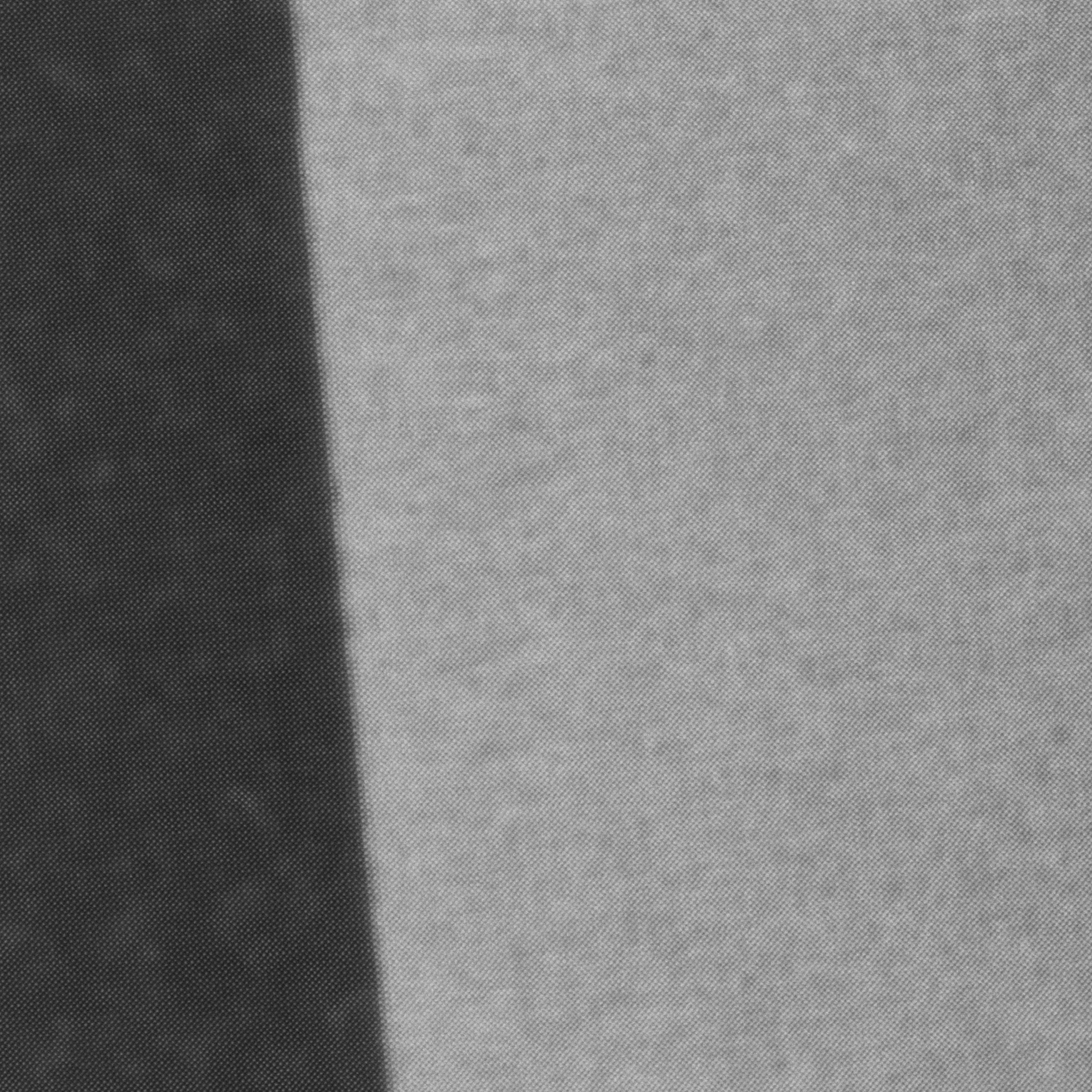

Supplement: Supplementary file 3 — Source Data [file 41467_2023_37117_MOESM3_ESM.zip › Source Data (Fig 2d).tif]

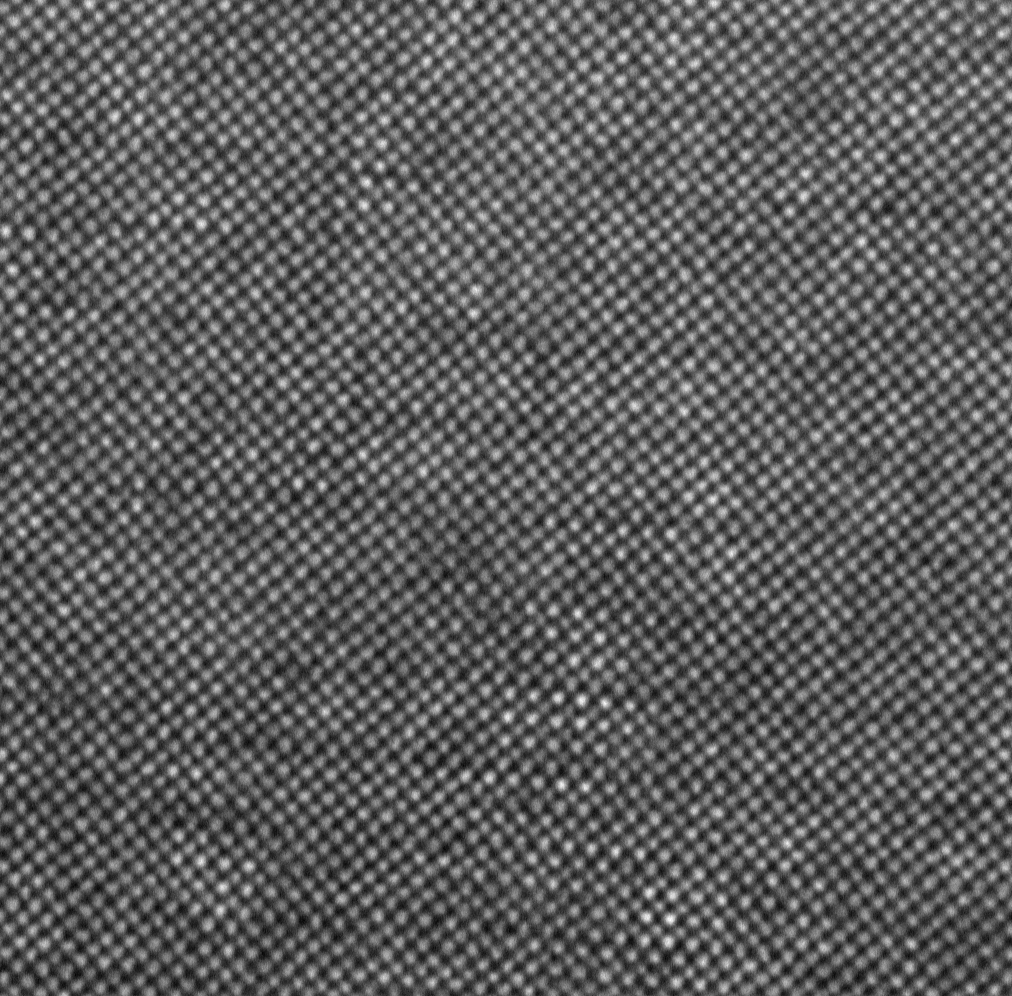

Supplement: Supplementary file 3 — Source Data [file 41467_2023_37117_MOESM3_ESM.zip › Source Data (Fig 2e).tif]

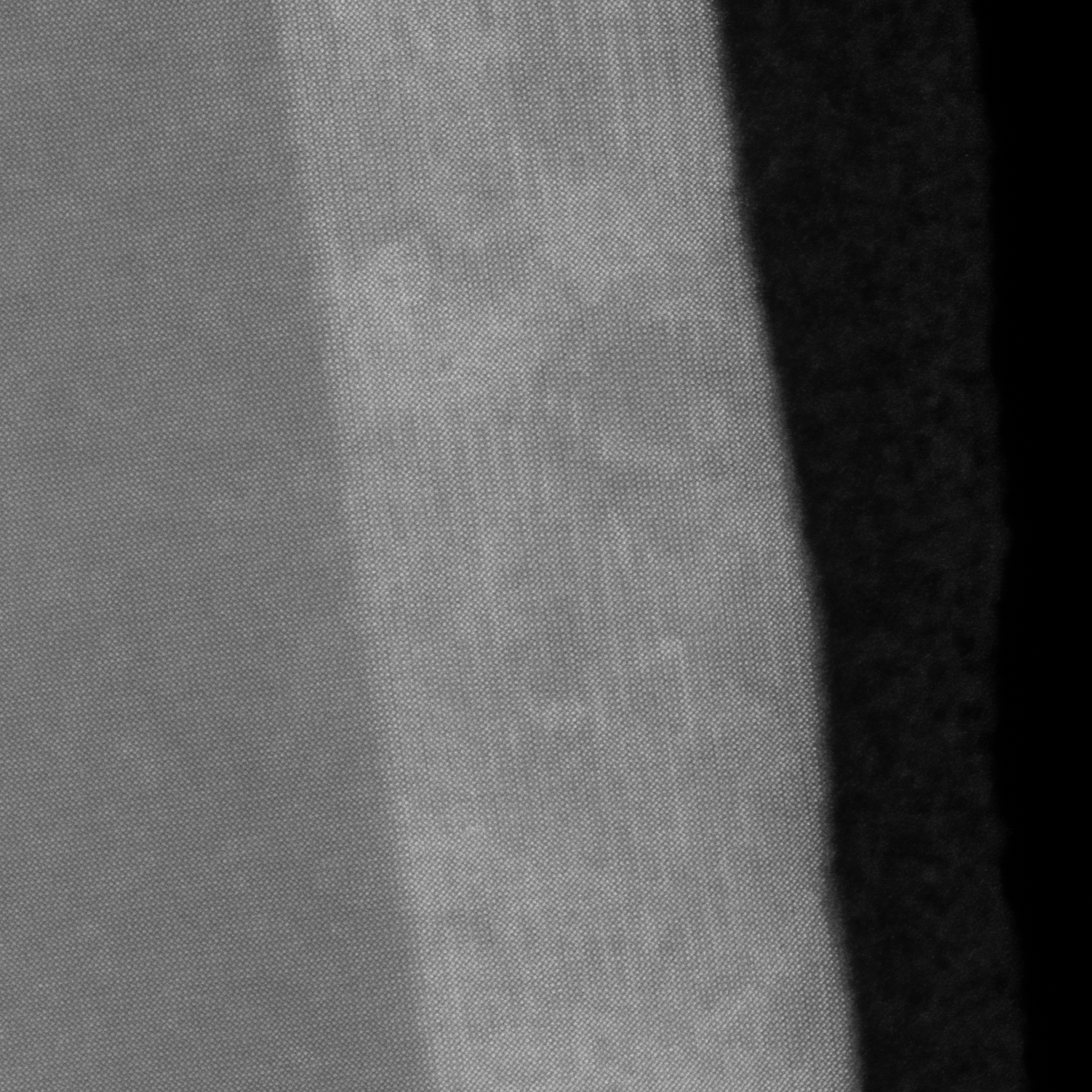

Supplement: Supplementary file 3 — Source Data [file 41467_2023_37117_MOESM3_ESM.zip › Source Data (Fig 3c).tif]

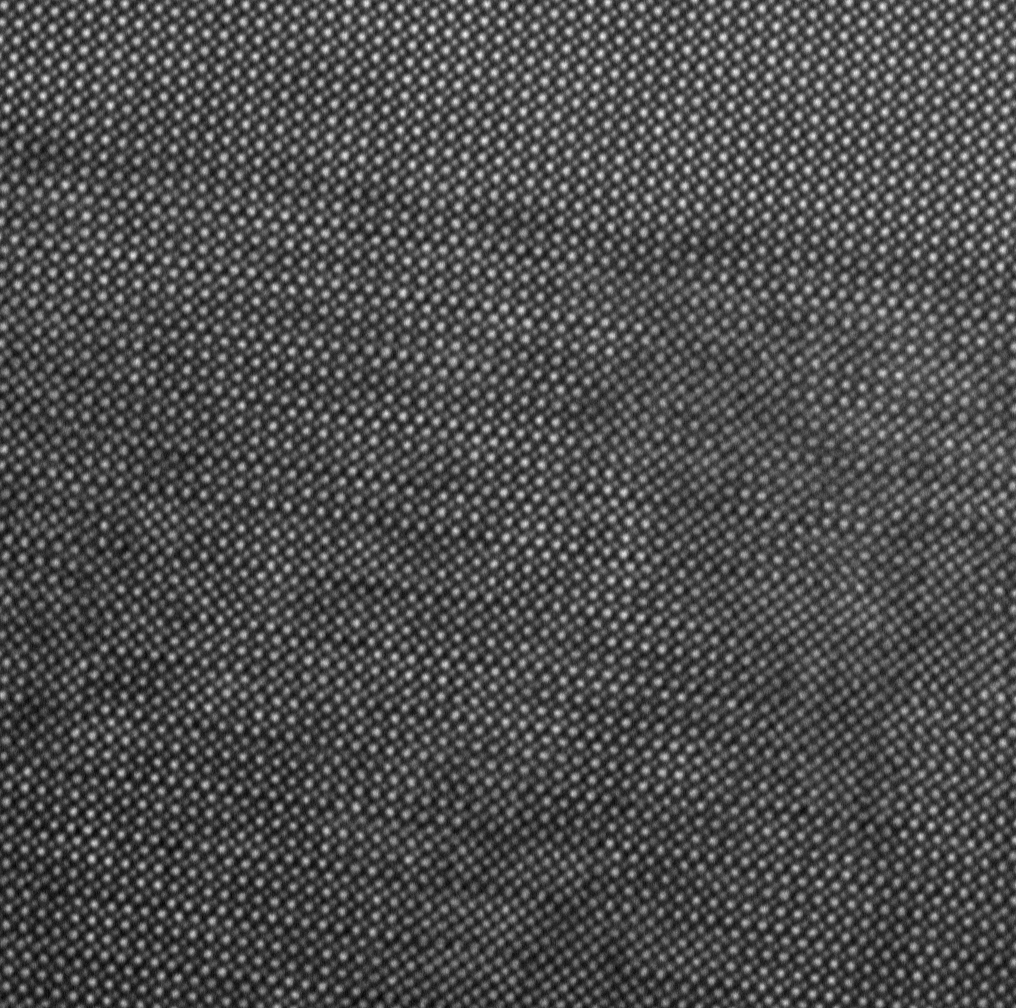

Supplement: Supplementary file 3 — Source Data [file 41467_2023_37117_MOESM3_ESM.zip › Source Data (Fig 3d).tif]

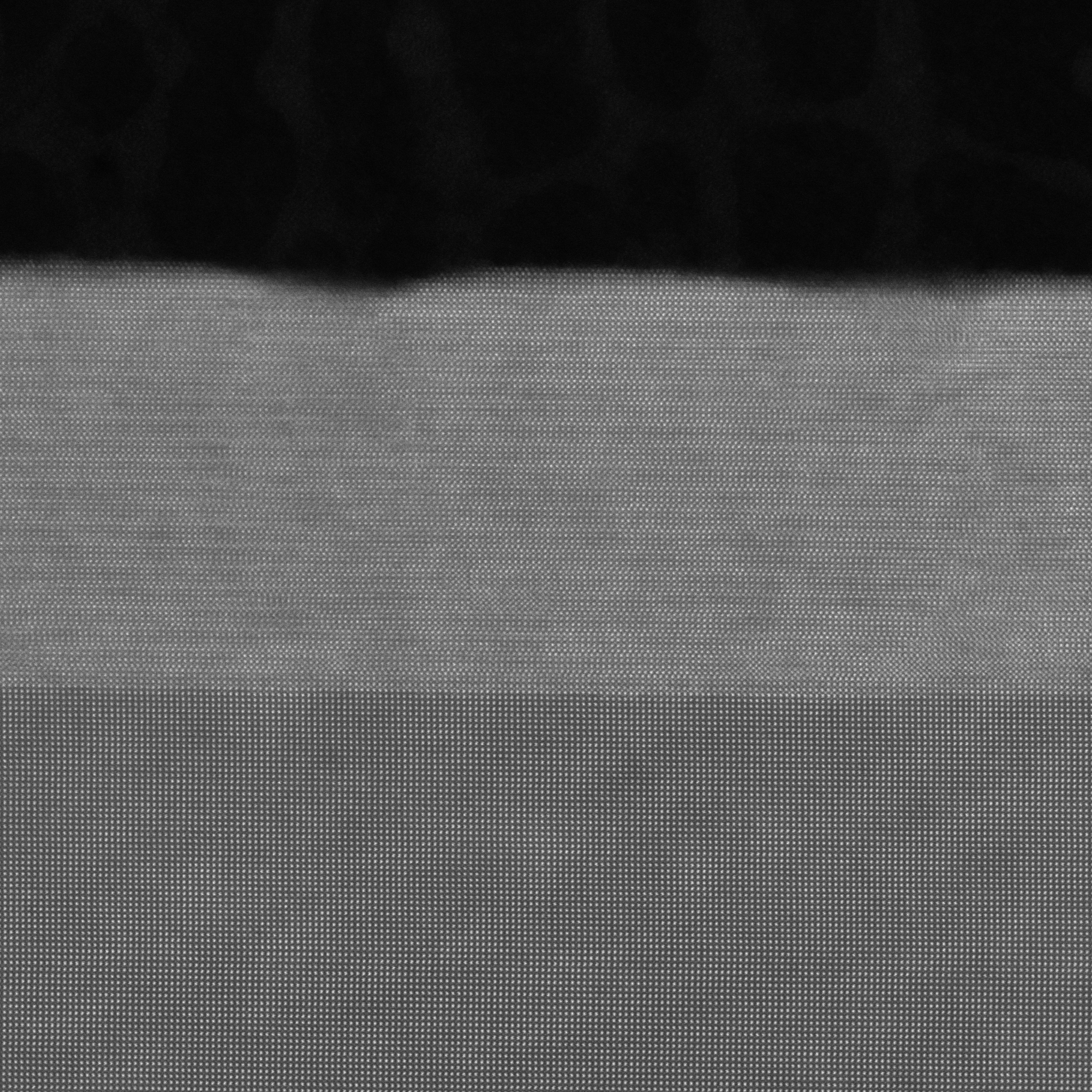

Supplement: Supplementary file 3 — Source Data [file 41467_2023_37117_MOESM3_ESM.zip › Source Data (Fig 4c).tif]

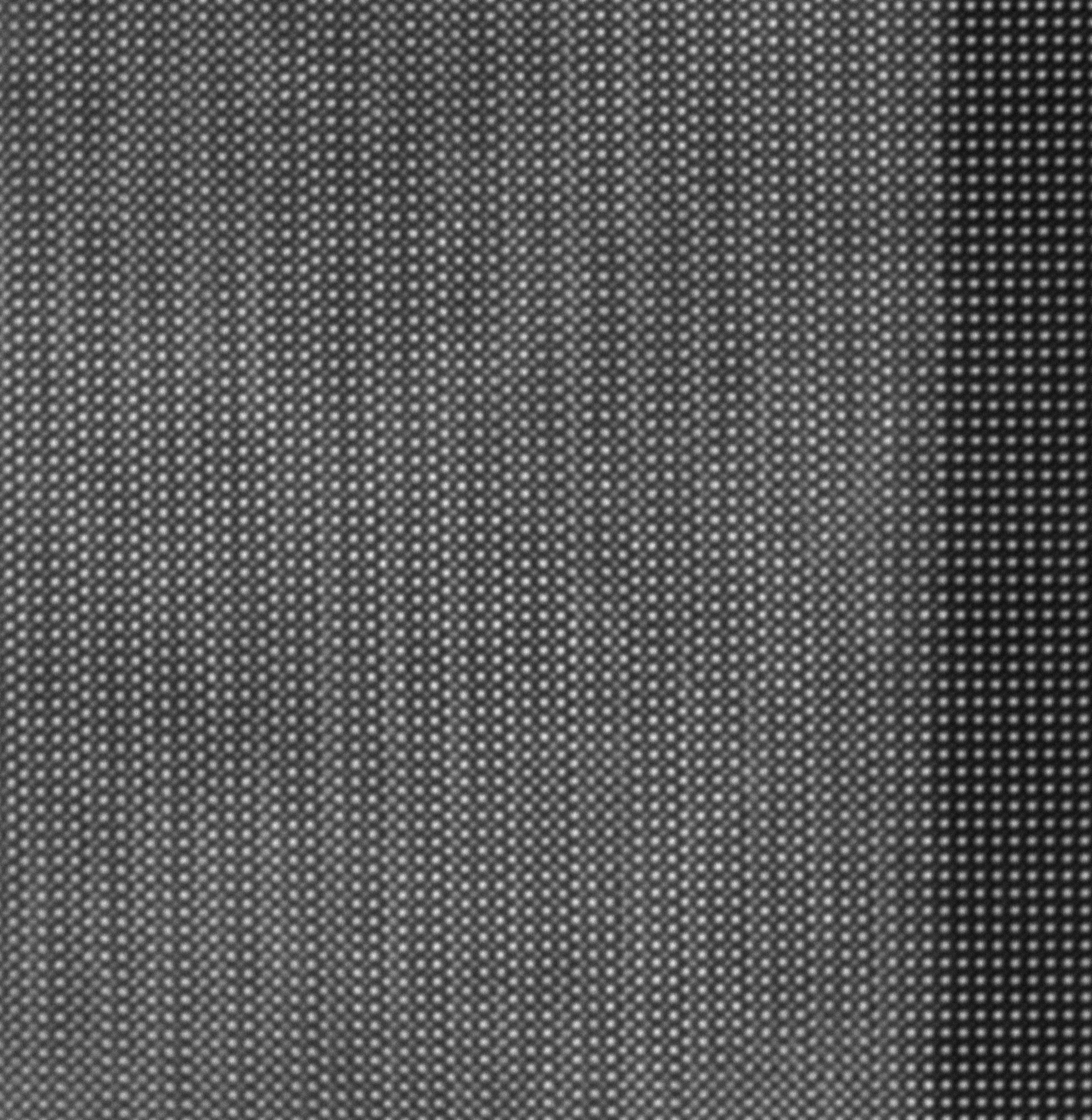

Supplement: Supplementary file 3 — Source Data [file 41467_2023_37117_MOESM3_ESM.zip › Source data (Fig 4d).tif]

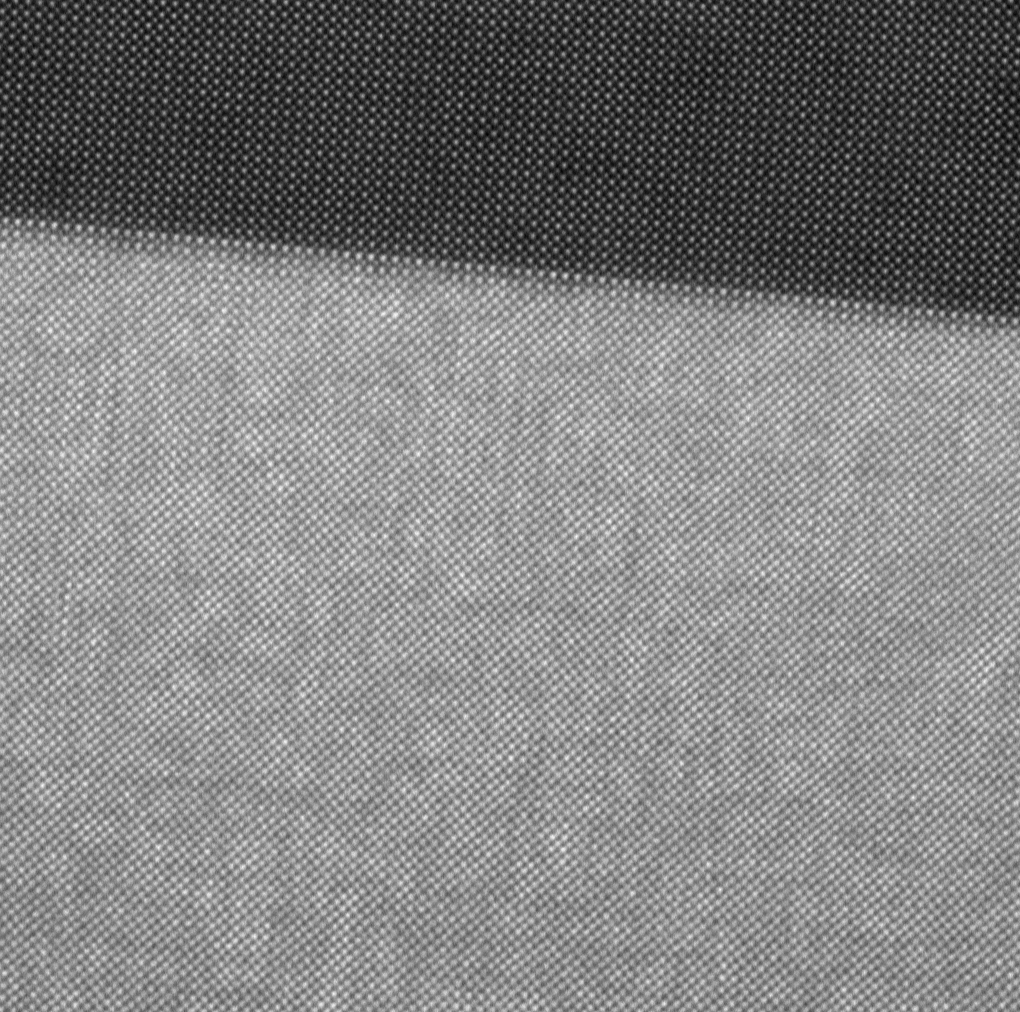

Supplement: Supplementary file 3 — Source Data [file 41467_2023_37117_MOESM3_ESM.zip › Source data (Fig 5a).tif]

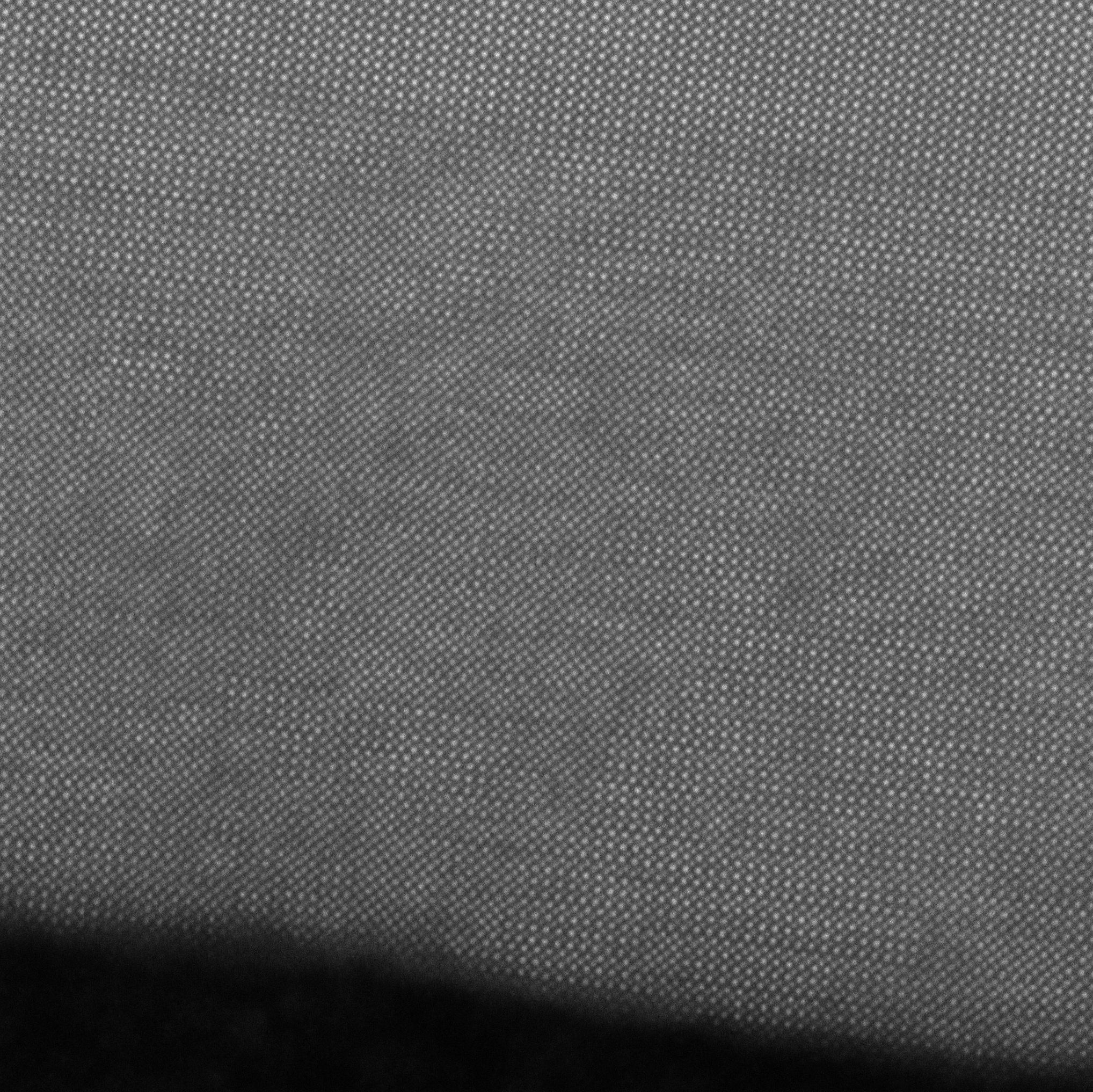

Supplement: Supplementary file 3 — Source Data [file 41467_2023_37117_MOESM3_ESM.zip › Source data (Fig 5b).tif]

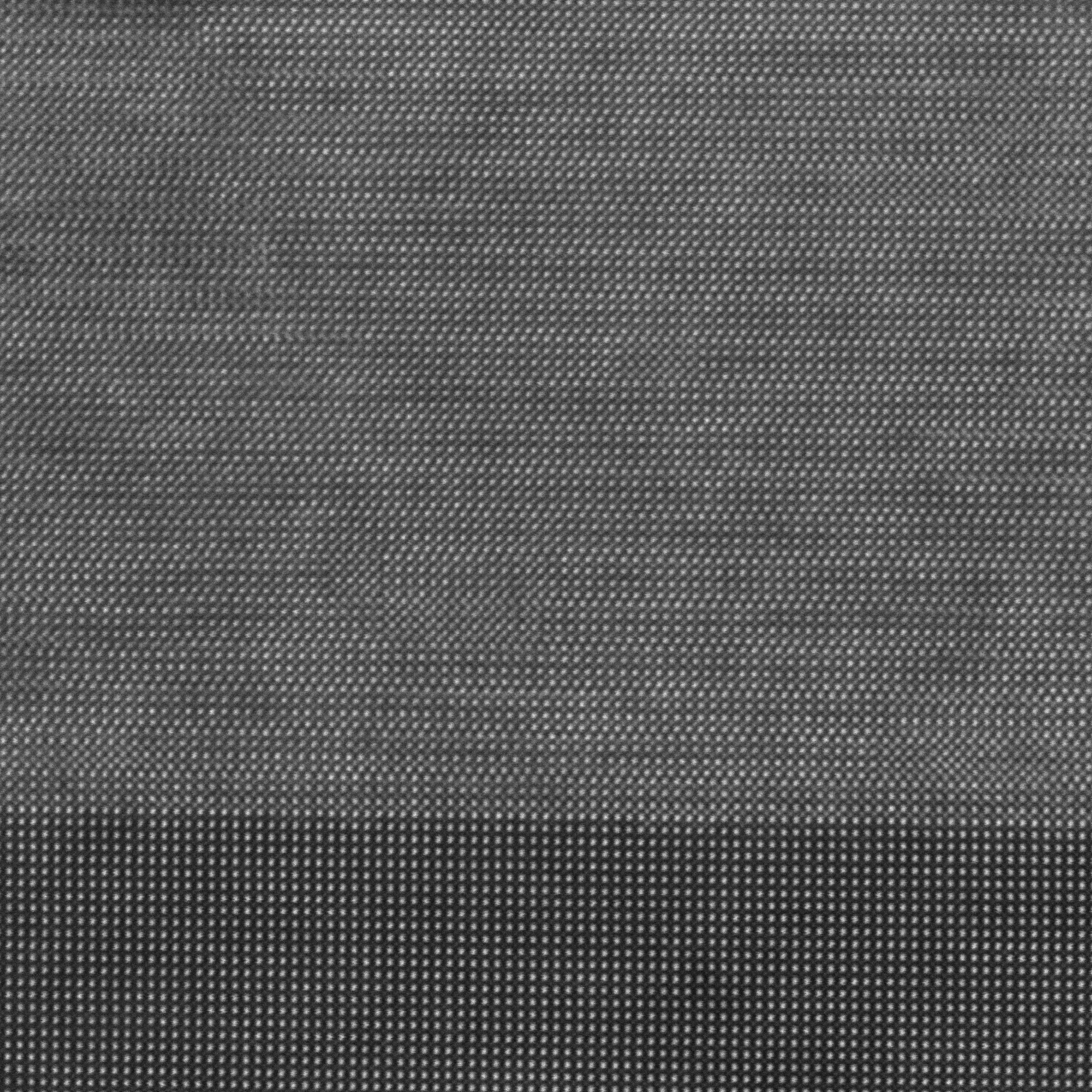

Supplement: Supplementary file 3 — Source Data [file 41467_2023_37117_MOESM3_ESM.zip › Source Data (Fig 5c).tif]

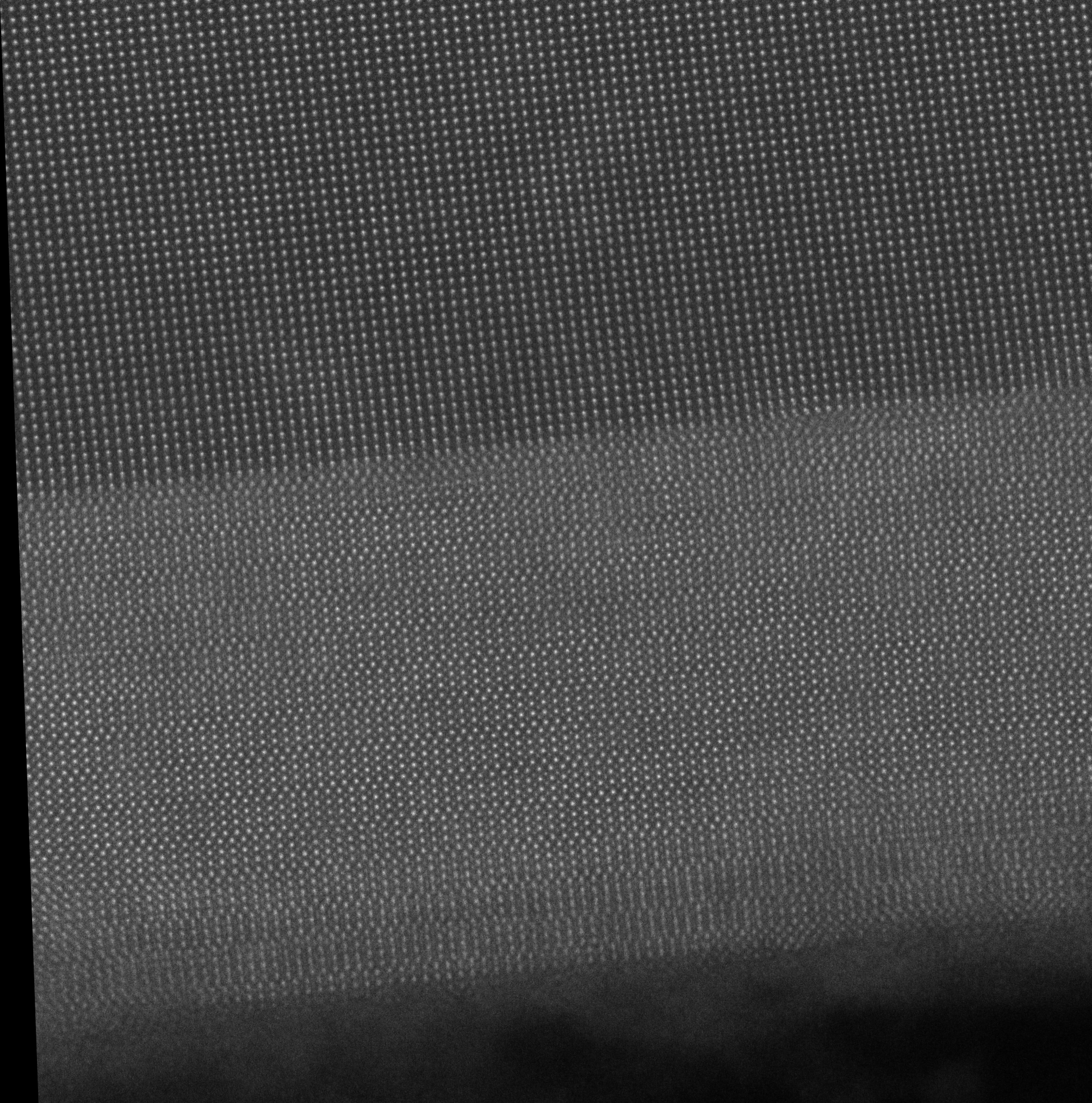

Supplement: Supplementary file 3 — Source Data [file 41467_2023_37117_MOESM3_ESM.zip › Source data (Fig 7b).tif]

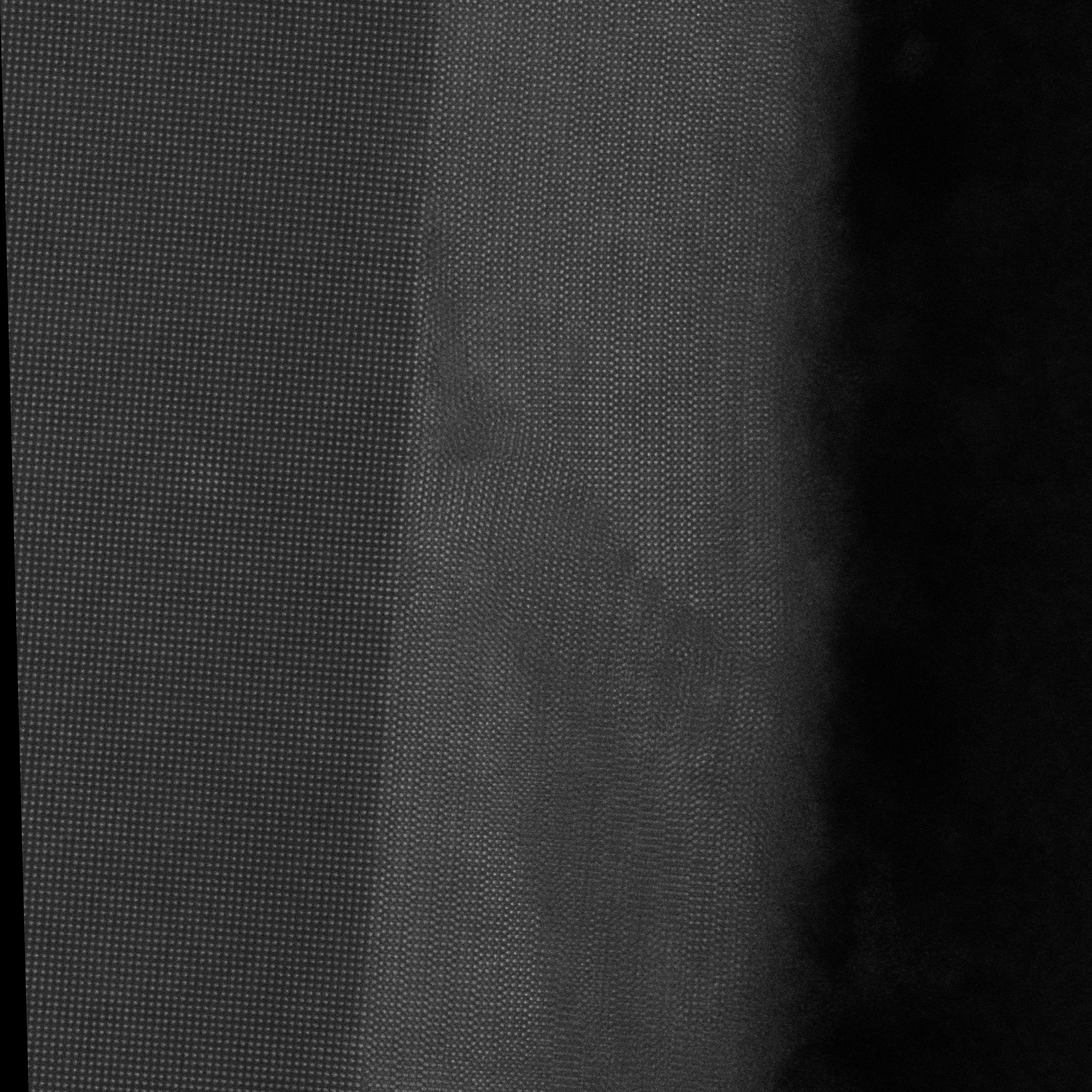

Supplement: Supplementary file 3 — Source Data [file 41467_2023_37117_MOESM3_ESM.zip › Source Data (Fig 7e).tif]

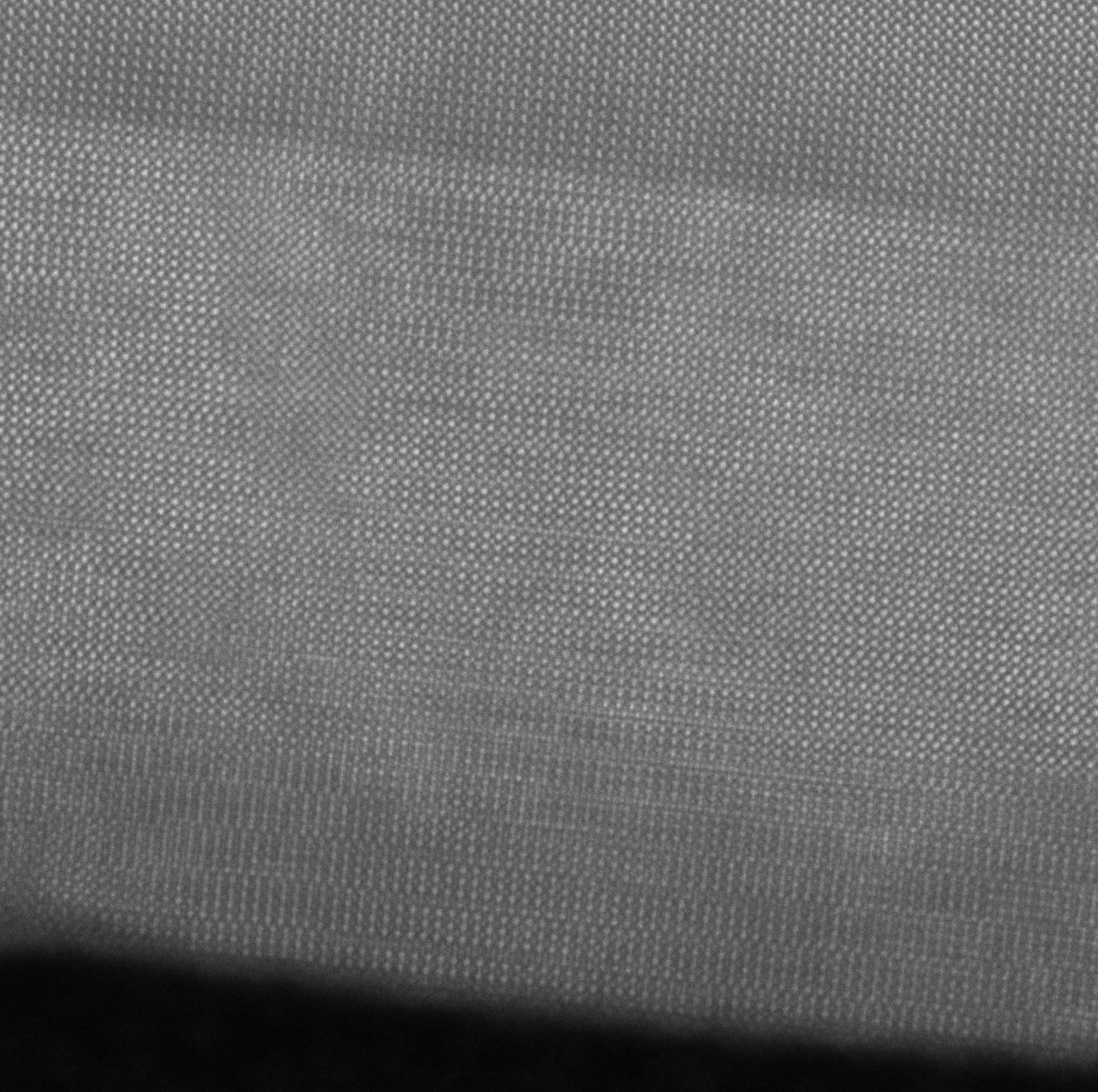

Supplement: Supplementary file 3 — Source Data [file 41467_2023_37117_MOESM3_ESM.zip › Source Data (Fig 9b).tif]

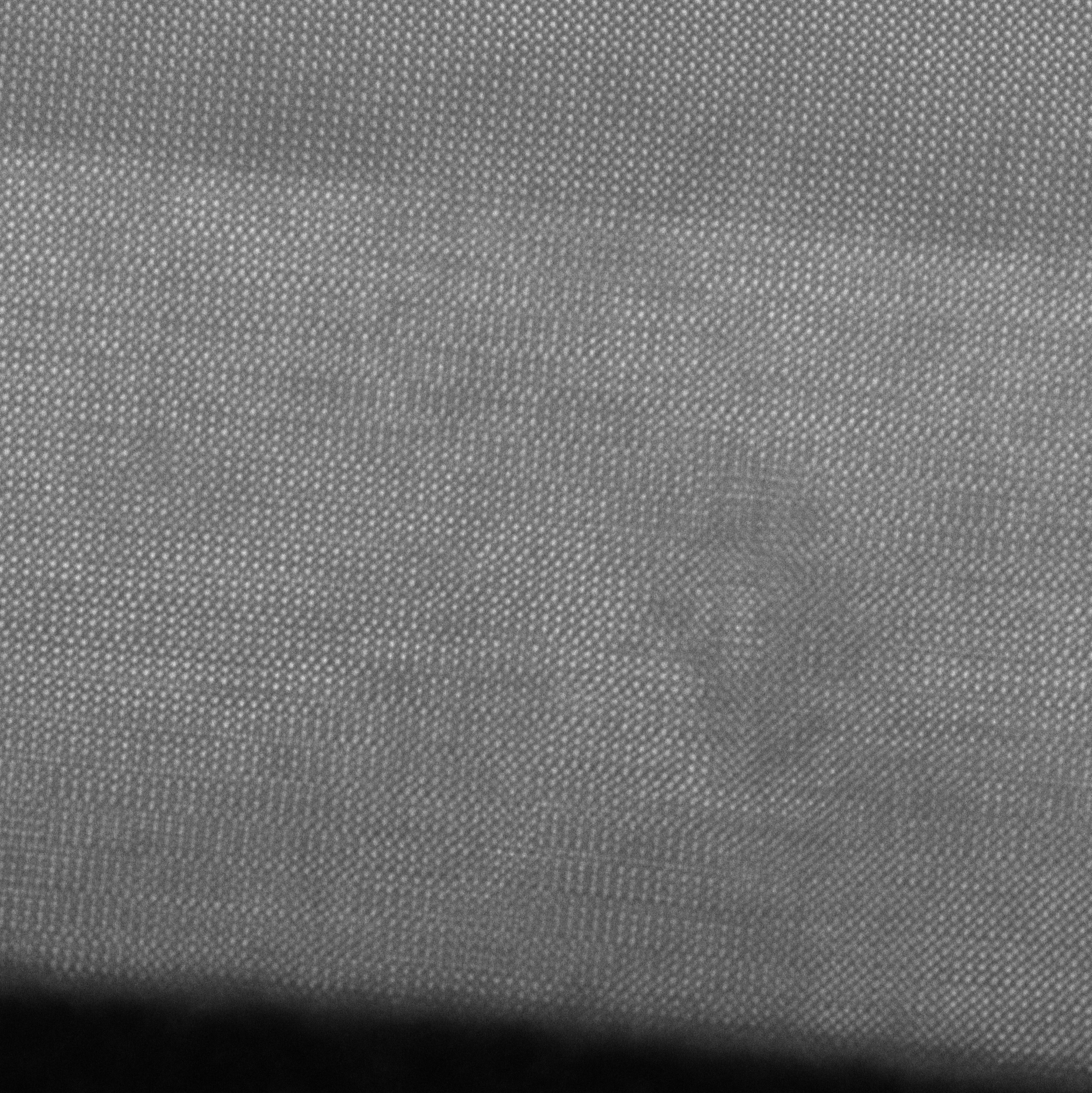

Supplement: Supplementary file 3 — Source Data [file 41467_2023_37117_MOESM3_ESM.zip › Source Data (Fig 9c).tif]

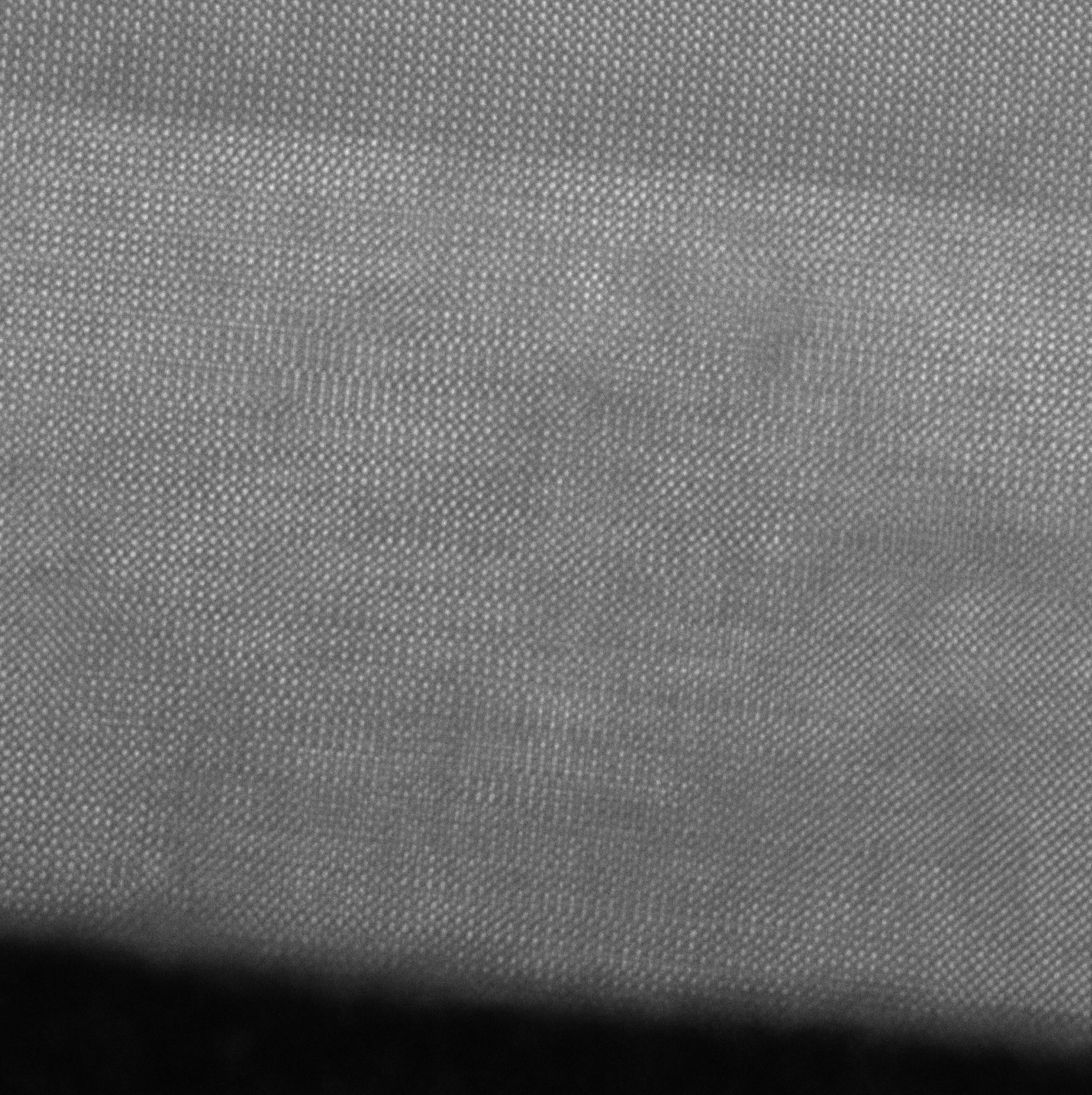

Supplement: Supplementary file 3 — Source Data [file 41467_2023_37117_MOESM3_ESM.zip › Source Data (Fig 9d).tif]
